# Polyetheretherketone for craniomaxillofacial defects: cases report, evaluation of patients’ satisfaction and a systematic literature review

**DOI:** 10.1186/s40902-025-00482-9

**Published:** 2025-10-24

**Authors:** Yuliya Menchisheva, Alvaro Varela Morillas, Nunzianda Frascione

**Affiliations:** 1 King’s College London, London, United Kingdom; 2 Kazakh National Medical University, Almaty, Kazakhstan

**Keywords:** PEEK, Reconstruction, Surgical procedures, Postoperative complications, Patient outcome assessment, Implant materials

## Abstract

**Background:**

Craniomaxillofacial reconstruction poses significant clinical challenges due to the complexity of the anatomy and the varied causes of defects. Selecting the optimal implant material remains a crucial factor in achieving successful functional and aesthetic outcomes.

This study combined a systematic review and a retrospective case series conducted at the Hospital 5, Almaty, Kazakhstan. The sample consisted of 52 patients who underwent craniomaxillofacial reconstruction between 2021 and 2024, receiving either PEEK, titanium, PMMA or silicone implants. Following the surgical procedures, patients were invited to participate in an online survey to evaluate their satisfaction with long-term outcomes.

**Results:**

PEEK implants demonstrated the complication rate at 22.2%, attributable only to hematoma. Titanium implants exhibited the complication rate—22.7%, with cases of asymmetry and diplopia (4.5%), exposure (9.1%), hematoma (4.5%) and infection with rejection (9.1%). Aesthetic outcome scores, measured by the ANA scale, varied across materials. PEEK implants achieved the highest mean ANA rating with 8.86 (SD = 0.35; 8.25–9.25), showing a significant difference over PMMA, silicone and titanium.

**Conclusions:**

PEEK implants demonstrated promising clinical and aesthetic outcomes in craniomaxillofacial reconstruction. However, material selection should be personalised, considering defect location, soft tissue coverage to optimise results.

**Supplementary Information:**

The online version contains supplementary material available at 10.1186/s40902-025-00482-9.

## Introduction

Reconstruction of defects in the maxillofacial region poses an ongoing challenge for clinicians. Different biomaterials such as titanium, autologous bone grafts and polymers are being used for facial defect reconstruction [1]. However, for larger and more complex defects, physicians are still struggling to find optimal solutions that can improve clinical and aesthetic outcomes and ensure patient satisfaction with the treatment results [2].

Facial bone defects can cause facial deformities, impairments in chewing and speech and have a profound impact on the physical and mental health of patients, seriously reducing their quality of life [3]. These defects and deformities in maxillofacial area could be congenital or acquired, resulting from traumas, resection due to benign and malignant tumours, odontogenic and non-odontogenic infections as well as osteonecrosis.

The main groups of alloplastic materials for facial plastic and reconstructive surgery include metals such as titanium, silicone, polymers like polyethylene, methylmethacrylate and polyetheretherketone (PEEK) and ceramics like hydroxyapatite [4]. Depending on the intended use of an implant materials’ (load-bearing or aesthetic region) the distinct qualities of the material should be carefully considered before their application [5].

Recent reports acknowledge that PEEK implants are an excellent treatment option and could effectively restore both bone and soft tissue deformities in facial surgery, meeting patients’ desires and requirements for reconstructive or cosmetic purposes [6–12].

PEEK is a polyaromatic semi-crystalline thermoplastic polymer which has all the mechanical properties suitable for bio-medical applications [13]. It is a lasting material which is widely used in different fields of medicine especially for surgical purposes including oral and maxillofacial, orthopaedic, spine, plastic and reconstructive surgeries. Its benefits within the field of reconstructive surgery have been significantly supported by various studies [9, 10, 14, 15]. Over the past decade, there have been several reports on the utilization of PEEK material in maxillofacial reconstructive surgery. These reports are based on the analysis of sequential clinical cases [2, 16–18]. However, further studies with larger cohorts and comparative analysis with alternative implant materials are needed to validate the accuracy of these findings.

The purpose of this study was to perform a comprehensive systematic review of the literature and to conduct a retrospective review of clinical cases to evaluate the various materials utilized for craniomaxillofacial reconstruction, comparing their advantages, disadvantages, clinical applications, mechanical properties, aesthetic outcomes and complications. Patient satisfaction with surgical outcomes was also evaluated in the long-term postoperative period through an online survey.

The authors of this study hypothesize that PEEK implants offer superior clinical and aesthetic outcomes in craniomaxillofacial reconstruction compared to titanium, polymethylmethacrylate (PMMA) and silicone, due to its favourable mechanical properties and biocompatibility. Further, it is proposed that careful patient selection and implant material choice will optimize outcomes and patient satisfaction.

The specific aims of this study were to (1) systematically review the literature on materials used for craniomaxillofacial reconstruction; (2) compare the advantages and disadvantages of widely used implant materials based on clinical applications, mechanical properties, biocompatibility, aesthetic outcomes and complications; (3) analyse a retrospective review of 52 cases of craniomaxillofacial reconstruction surgeries to share insights from past clinical experience, including surgical outcomes and complications; and (4) evaluate patient satisfaction with surgical outcomes in the long-term postoperative period through an online survey.

The findings from this study contribute to the growing body of evidence regarding material selection in craniomaxillofacial reconstruction. By combining a systematic review of the literature with clinical data and patient satisfaction surveys, this research provides a multifaceted evaluation of implant materials.

## Material and methods

The protocol for this study was registered in PROSPERO (ID: CRD420251079557, https://www.crd.york.ac.uk/PROSPERO/view/CRD420251079557) and compiled by the Preferred Items for Systematic Reviews and Meta-Analyses (PRISMA) reporting guidelines [19]. The study selection process is illustrated using the PRISMA flow chart [20]. Supplemental materials have more details on the search methodology.

### Eligibility criteria

Specific inclusion and exclusion criteria to ensure the selection of relevant studies were established (Supplemental Table 1).

**Table 1:** Main advantages and disadvantages of the most common materials for reconstructive surgery

Material	Advantages	Disadvantages
Titanium	Excellent biocompatibility, corrosion resistance, high mechanical strength, osseointegration, not ferromagnetic, durable, stable, rigid	Thermal sensitivity, not translucent—cause radiographic distorge, cosmetic issues, potential for allergic reactions (as a result lead to rejection)
Silicone	Suitable for aesthetic purposes, flexible, easy removal due to encapsulation	Bone resorption, lack of osseointegration, high infection and displacement rates, high rate of asymmetry due to displacement, high removal rate, poor integration
Polyethylene	Allows tissue ingrowth, availability	Thermal sensitivity, hard to remove, high risk of infection and extrusion, lack of rigidity (not ideal for load-bearing areas)
PMMA	Radiolucent, customizable before polymerization, biocompatible, bone ingrowth in porous form implants, cost-effective	Provide exothermic reaction, difficulty in reshaping after polymerization, lack of osseointegration (non-porous forms), high risk of infection (for porous implants), not load-bearing
Bioceramics (HA)	Promotes cellular adhesion and osseointegration, excellent biocompatibility, osseoconductivity, replaced by newly formed bone, low immune rejection risk	Brittle, low mechanical strength, prone to fracture, potential infection, less suitable for load-bearing areas
PEEK	High strength and stiffness, radiolucent, non-magnetic, resistant to gamma radiation, no thermal reactions and thermal sensitivity, low weight, similar flexibility to bone, steam sterilization	Bio-inert, requires surface modification, poor adhesion, incidence of infection, higher cost, limited long-term data

### Search strategy

The Population, Intervention, Comparison, Outcome (PICO) framework was applied to ensure a structured and systematic approach to formulating the research question and guiding the literature search.

#### PICO framework

- Population (P): Patients with maxillofacial defects who underwent reconstructive surgery.
- Intervention (I): Use of PEEK implants for reconstruction.
- Comparison (C): Other implant materials such as titanium, polymethylmethacrylate (PMMA), silicone, polyethylene, hydroxyapatite.
- Outcome (O): clinical outcomes (implant success rates, complication rates), patient satisfaction, functional outcomes (improvement in chewing, speech and overall quality of life).

A literature search for the review of facial implant materials in maxillofacial surgery was conducted through MEDLINE (PubMed), EMBASE and The Cochrane Library databases. All human studies published in the English language from the year 2000 onwards reporting on implants used in facial reconstruction were considered eligible for inclusion. The electronic search strategy was based on specific inclusion criteria, utilizing a combination of Medical Subject Headings (MeSH) and entry terms. The following search terms were applied: (“PEEK implants” OR “polyetheretherketone implants” OR “titanium implants” OR “PMMA implants” OR “silicone implants” OR “polyethylene implants” OR “hydroxyapatite implants”) AND (“maxillofacial reconstruction” OR “facial bone defects” OR “craniofacial reconstruction”) AND (“patient satisfaction” OR “clinical outcomes” OR “aesthetic outcomes” OR “complication” OR “functional outcomes”). According to the inclusion and exclusion criteria, the articles were chosen based on their titles and abstracts (Appendix A).

### Selection process

All the studies retrieved were screened in two stages using Rayyan software for systematic review. First, two independent reviewers evaluated the titles and abstracts to determine relevance. Then, full texts of the selected studies were reviewed, and only those meeting the inclusion and exclusion criteria were considered eligible for analysis.

### Data collection process

EndNote, a reference management software, was used to streamline the data collection and organisation process. Duplicate articles were removed. The articles were selected by title and abstract for relevance according to the specified inclusion and exclusion criteria outlined below. All abstracts were independently reviewed. Any disagreements were resolved by the third author's involvement (AM) [21]. Google Scholar Alerts was used to update citations extended to 31st March 2025 in order to ensure the inclusion of the most recent and relevant publications.

### Study risk of bias assessment

The risk of bias assessment was conducted using two validated tools based on study design. The Joanna Briggs Institute (JBI) critical appraisal checklist (Australia, JBI) was used for case reports and case series. For retrospective and prospective studies, the Risk of Bias in Non-Randomised Studies of Interventions (ROBINS-I) (UK, Cochrane) was applied to assess potential biases in studies.

### Selection criteria

The inclusion and exclusion criteria for study eligibility were established in the study protocol. The current evaluation covered every kind of clinical investigation involving human participants, including controlled clinical trials, prospective and retrospective studies, case series and case reports. On the other hand, review papers and experiments conducted in vitro or on animals were not included. The records where the titles and abstracts included the words “dental”, “prosthetic rehabilitation”, “use of injectable implants/fillers”, literature reviews without clinical cases, systematic reviews, meta-analysis, in-vitro studies and animal studies were excluded.

### Retrospective study

Data from medical records of all 18–74 years old patients who underwent maxillofacial reconstruction procedures using different implant materials such as PEEK, titanium, PMMA and silicone at Hospital 5 in Almaty, Kazakhstan, between 2021 and 2024 were collected. The informed consent for the use of patient data was obtained through an ethical process. The hospital administrator initially contacted eligible patients via phone, explaining the study’s purpose and requesting verbal consent for the retrospective analysis of existing medical records. The study protocol was approved by the Research Ethics Committee of King’s College London (Ref. number LRS/DP-24/25–4531 from October 3, 2024). Data such as age, sex, diagnosis, localization of the bone defect or deformity, cause of the bone defect or deformity, material of the implant that was used for reconstruction, type of reconstruction (primary or delayed), duration of operation in minutes, number of occupied bed days, postoperative complications, comorbidities, bad habits, presence of preoperative or postoperative radiotherapy or bisphosphonate medication were collected. All data were anonymised. Assessment of the incidence of complications, such as infection, hematoma, implant failure and others, following facial reconstructions using PEEK, titanium, PMMA and silicone implants was also provided.

#### Surgical technique

All surgeries were performed under general anaesthesia using standardised protocols by the same surgical team. Implant fit was pre-planned using CAD/CAM (Computer-aided design and manufacturing) design and patient-specific 3D models. All implants were customised and fabricated according to defect location and contour. PEEK implants were fixated using self-tapping titanium screws (1.5–2.0 mm), with bicortical engagement where possible, typically in 3–4 quadrants. Titanium implants fixation followed standard osteosynthesis principles. PMMA implants were prefabricated preoperatively and sterilised using ethylene oxide. Implants were seated into the defect and secured with titanium screws to the surrounding bone. Silicone implants were preformed and positioned in subperiosteal pockets. Fixation was performed using titanium microscrews. Hemostasis was carefully achieved using bipolar cautery and absorbable hemostatic agents. In all cases, layered wound closure was performed with resorbable 3–0, 4–0 or 5–0 sutures. A closed-suction drain was placed where indicated and removed after 24–48 h.

#### Postoperative protocol

Postoperative care included antibiotics (ceftriaxone 1 g IV for 5 days). Analgesia was administered via NSAIDs. Wound care involved sterile dressing changes every 24 h until suture removal (at day 7–10). Patients were monitored in the inpatient ward for an average6.13 ± 0.23 days. All patients underwent standardised clinical follow-up at 1 week, 1 month, 3 months and 12 months postoperatively. Complications were assessed during each follow-up period by the same clinical team using a uniform checklist.

### Aesthetic outcomes

The study included an assessment of the aesthetic outcomes of patients who already underwent surgical procedures using different implant materials for maxillofacial defects and deformities at Hospital 5 in Almaty, Kazakhstan, between the years 2021 and 2024.

**Fig. 1:**
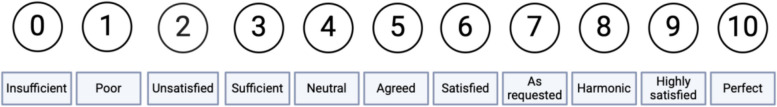
Aesthetic numeric analogue scale. Created with BioRender.com

The survey was conducted anonymously using a structured questionnaire. The questionnaire was designed using the Google Forms application to assess the patient’s satisfaction with the aesthetic outcome of their surgery. For the online survey, additional consent was obtained. All participants received the information sheet and consent form via WhatsApp. A series of questions rated on an eleven-point scale according to the “Aesthetic Numeric Analogue” (ANA) were included (Fig. 1). Patients were asked to select the word that best described their level of satisfaction with the aesthetic results of their reconstructive surgery.

The responses from the questionnaires were collected and coded numerically, with each descriptor assigned a specific score. The survey was conducted in accordance with the ethical standards of the committee responsible for human experimentation (institutional and national), and with the Helsinki Declaration of 1975, as revised in 2013 (http://ethics.iit.edu/ecodes/node/3931). Written consent was obtained from all parents/legal guardians of participants enrolled in the study.

### Statistical analysis

Descriptive statistics summarised the patient characteristics and outcomes. Chi-square tests compared the complication rates, while the Kruskal–Wallis test was used to analyse aesthetic outcomes across the four implant materials (PEEK, titanium, PMMA, silicone).

To evaluate the adequacy of the sample size for detecting differences in complication rates among the four implant material groups, a post-hoc power analysis was conducted using G*Power 3.1. software. The analysis assumed an effect size f = 0.35 (medium-to-large), α = 0.05, and power 1–β = 0.80. The observed effect sizes were computed and interpreted with corresponding 95% confidence intervals. Additionally, power calculations were repeated using smaller effect sizes (f = 0.20–0.25) to determine the minimum detectable differences under more conservative assumptions.

## Results

### Systematic literature review

**Fig. 2:**
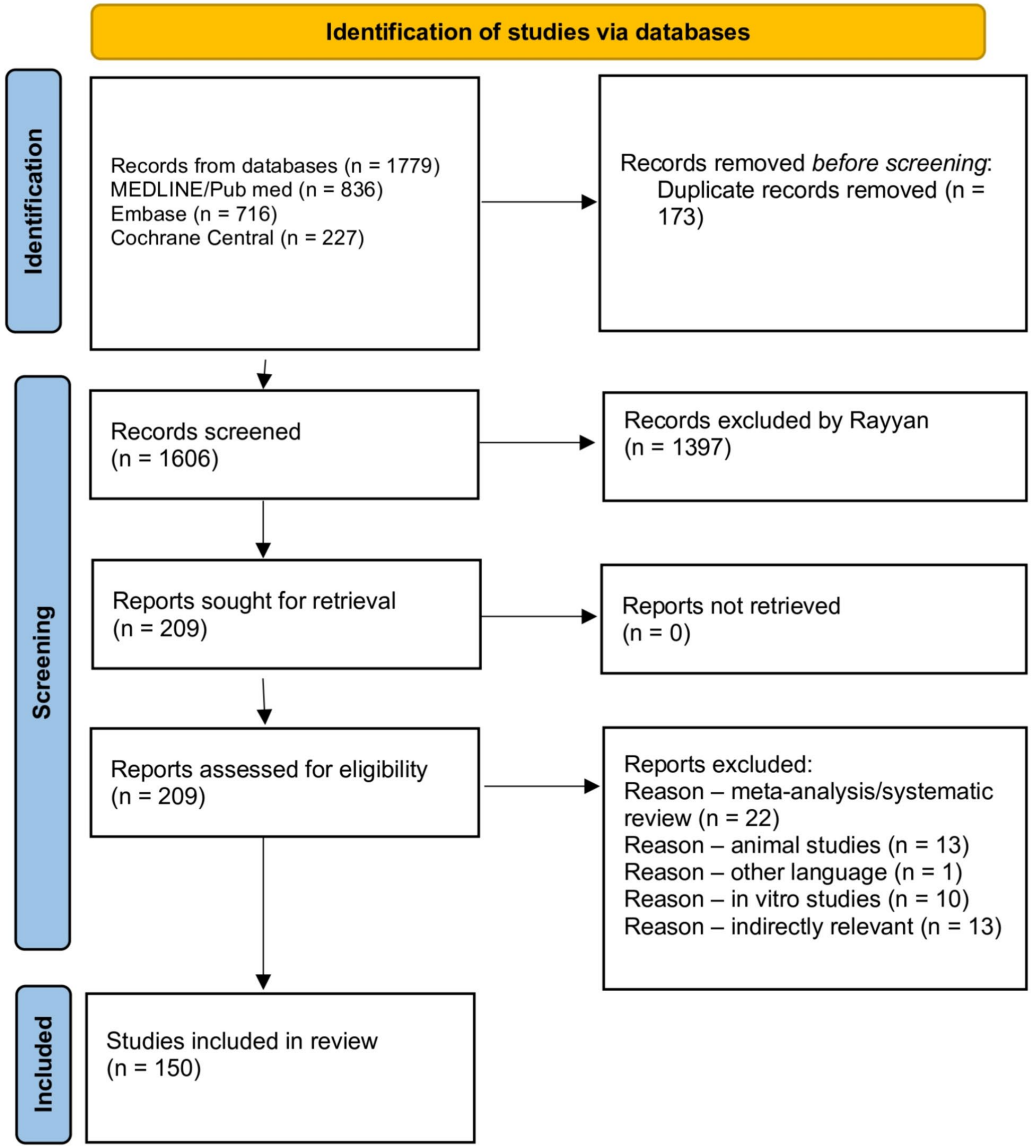
The PRISMA flow chart. Created with Github.com

There were 3685 patients in 150 studies overall. There were 42 studies (with 821 patients) in the titanium group, 12 studies (with 806 patients) in the silicone group, 24 studies (with 914 patients) in the polyethylene group, 18 studies (with 382 patients) in the acrylic material—PMMA group, 41 studies (with 479 patients) in the PEEK group, 13 studies (with 283 patients) in the hydroxyapatite group. The PRISMA flow diagram is illustrated in Fig. 2.

#### Titanium

Titanium is widely used in reconstructive maxillofacial surgery, particularly for zygomatic-orbital defects, due to its high mechanical strength, corrosion resistance and biocompatibility [22]. The main advantages of titanium implants are their durability, stability, rigidity and ability to provide osseointegration [13, 23]. However, titanium’s extreme rigidity limits its use in aesthetic augmentation. Its thermal sensitivity can result in an uncomfortable sensation of cold that frequently leads to removal due to the implant’s higher heat conductivity than the surrounding tissues [24]. It also produces imaging artifacts and carries risks of implant rejection [25]. Studies also highlighted the challenges in precise reconstruction with titanium [26]. Pietzka et al. [27] reported a 66.6% inaccuracy rate in orbital reconstructions using patient-specific titanium implants. In the zygomatic-orbital region, implant exposure has been observed due to thin overlying soft tissue, compromising cosmetic results [13, 28–30]. Titanium nasal implants, introduced in 2003, aimed to improve airway patency [31]. Titanium meshes and plates are often used for structural support in reconstructive surgery and to replicate maxillary or mandibular contours [32, 33]. Mounir et al. [34] documented three failures out of seven cases using patient-specific titanium endoprostheses for mandibular reconstruction, due to mucosal dehiscence and fistula formation. In contrast, Woo et al. [35] demonstrated successful contour restoration of the mandibular angle and its lower border using titanium implants. Although titanium is corrosion-resistant, allergic reactions have been reported [36–38].

#### Silicone

Silicone is widely used in facial skeleton implants especially for aesthetic purposes. Silicone implants can be premanufactured (i.e. malar, chin, mandibular implants) or a silicone block can be used, from which the surgeon cuts out the required sized implant. It is worth to acknowledge that this material has higher incidence of infections and displacements, depending on the implanted area [39]. The chin and mandibular area are considered the safest, while malar implants have a high incidence of prominence [40]. However, Al-Jandan et al. [41] reported cases of displacement of silicone implants in mandibular angle augmentation. According to a recent narrative review by Gafar Ahmed et al. [39], silicone implantation has a range of complications such as asymmetry, bone resorption or erosion, displacement, dissatisfaction, oedema, hematoma, infection, mucosal irritation, pain and paraesthesia. Over time, the substance usually becomes encapsulated by a fibrous membrane and is not integrated into the host. This makes removal easier but needed due to seroma formation, extrusion, undesired implant displacement and poor cosmetic results [25]. In comparison to porous polyethylene and hydroxyapatite, silicone implants had one of the highest removal rates according to Rubin et al. [42]. It is worth acknowledging that the use of silicone implants is popular among cases of augmentation rhinoplasty. Despite its benefits, using silicone implants can lead to negative outcomes, including contracture deformity, immediate and delayed infection and an improper implant contour necessitating revision surgery [43]. Poor implant location and selection may be the cause of most malformations. The replacement of existing grafts with specially carved silicone implants achieves the desired aesthetic results [44]. The utilization of cheek silicone implants has proven to be an excellent technique for rejuvenating midfacial volume. The main challenge in this operation lies in choosing the right type, shape and size of the silicone implant [45].

#### Polyethylene

As an alternative to bone grafts, polyethylene is a biocompatible, porous, high-density implant material. It is commonly used in aesthetic plastic surgery for contour enhancement in cases of rhinoplasty, chin, mandibular and malar augmentation. In addition, polyethylene is utilized in congenital malformations, closure of post-traumatic defects and reconstruction after tumour removal [46]. Significant vascular and soft-tissue ingrowth is made possible by its porous nature. Soft tissue ingrowths, collagen deposition and eventual vascularization were confirmed by biopsies in a recent study by Niechajev [47].

One of the most popular applications in the use of polyethylene is auricular reconstruction for congenital malformations. The synthetic polyethylene material called Medpor is currently used to replace missing face cartilage, although it is not a suitable substitute for complex defects reconstruction due to its high rate of surgical complications, such as infection and extrusion [48]. Also, porous polyethylene implants are widely used with rib cartilage for microtia reconstruction. According to Ku et al. in their study, 5 (15.2%) of 33 hybrid frameworks cases (polyethylene + rib) were removed due to infection or extrusion, which is considered as a high explantation rate [49]. However, according to different recent research, nasal and paranasal augmentation using porous polyethylene is a dependable technique that causes the least amount of morbidity in patients with soft tissue insufficiency [50, 51].

#### Polymethyl methacrylate

Polymethyl methacrylate (PMMA) has been applied to numerous cases of orbital [52], fronto-orbital [53, 54], zygomatic reconstruction and cranioplasty [55, 56], midface and mandibular augmentation [57]. PMMA is a biocompatible and nondegradable acrylic resin-based implant [58]; and it also presents radiolucent properties, which makes it hard to detect in radiographs but benefits in ease post-operative imaging in the follow-up period [59]. PPMA implants are encapsulated during the time with fibrous connective tissue. If pathology recurs and revision surgery is necessary, this guarantees a straightforward removal [60].

The initial flexibility of the material can be attributed to the combination of the liquid polymer and the monomeric powder. Eventually, the substance undergoes an exothermic reaction and solidifies into a highly resilient material, achieving the desired shape [61]. Bruens et al. [56] reported long-term results of using the porous form of PMMA in patients with craniofacial defects over 20 years. An aqueous, biodegradable carboxymethylcellulose gel was dispersed to create pores in implants. PMMA was prepared using the traditional bone cement composition of methylmethacrylate liquid and PMMA powder. The pores provided space for bone resorption improving prosthesis fixation. Jain et al. [57] believe that patient-specific PMMA implants have become the gold standard for reconstructing cranial abnormalities, with outstanding long-term outcomes. However, PMMA was shown to have a relative rate of hematoma formation and infection following implantation, according to a meta-analysis of facial implant materials [62]. On the other hand, the meta-analysis conducted by Leao et al. [63] indicated that the use of PMMA yields complication rates near those of autologous bone and titanium mesh.

PMMA material is not usually used for midfacial augmentation or reconstruction, especially in load-bearing areas, because of the difficulty in reshaping it, the risk of infections and plate fractures and the lack of osseointegration [13].

#### Bioceramics

Recent studies have demonstrated that hydroxyapatite (HA)-based implants outperform other alloplastic implants in terms of promoting cellular adhesion and integration [64–67]. HA plates are used for grafting facial bones, such as zygoma, maxilla and mandible. The remarkable bio-acceptance of HA for facial augmentation was confirmed by the authors’ discovery that the HA granules are enveloped by host collagen and gradually replaced by neo-bone, including osteoblasts and osteocytes [68]. Another main advantage of using HA implants is their biocompatibility. As HA is a naturally occurring mineral found in human bones, there is a lower risk of immune rejection or adverse reactions compared to other materials [65]. This also means that the implant is less likely to cause inflammation or infection. However, it works well when used in the form of plates and meshes, while solid implants are significantly brittle. Researchers have reported that HA implants have a lower mechanical strength and toughness than titanium and PEEK implants, which make HA implants more prone to fracture or failure under mechanical stress, especially in load-bearing areas such as the mandible and maxilla [69]. Zhang et al. [70] emphasised the lack of confidence on the optimal balance between strength and osseointegration.

#### Polyetheretherketone

The use of PEEK implants in maxillofacial reconstructive surgery has gained significant popularity in recent years due to its many advantages over other implant materials. Commercialized for industry in the 1980 s [71], PEEK first described in cranioplasty by Hanasono et al. and has gained popularity since the early 2000 s [72]. According to Ma et al. [73], PEEK contains an aromatic molecular backbone and functional groups located between the acrylic rings that are a mix of ether and ketone.

PEEK has superior mechanical properties including high strength, stiffness and toughness which make it an ideal material for load-bearing applications [7, 15, 74]. When compared to alternative alloplastic implant materials, PEEK offers a number of benefits. PEEK does not cause artifacts on radiographic imaging and exhibits radiographic translucency, greatly enhancing picture quality, making it easier to radiologically analyse the tissues surrounding the implant [75]. This is advantageous for CT-based adjuvant radiation planning and radiological tumor follow-up [76]. In addition, PEEK does not undergo exothermic reactions like methyl methacrylate and is neither allergic nor magnetic [77]. Moreover, PEEK's flexibility is similar to that of cortical bone [73]. However, PEEK’s bio-inertia causes challenging osteogenesis and weak osteoconductive qualities [78]. To improve its osteoinductive and antimicrobial capabilities, different types of functionalization of PEEK surfaces and changes in PEEK structure have been proposed by various authors [79, 80]. In contrast, PEEK implants are a relatively new material and have not been extensively studied in long-term clinical trials.

The advantages and disadvantages of the six discussed implant materials used in reconstructive maxillofacial surgery are summarised in Table 1.

**Fig. 3:**
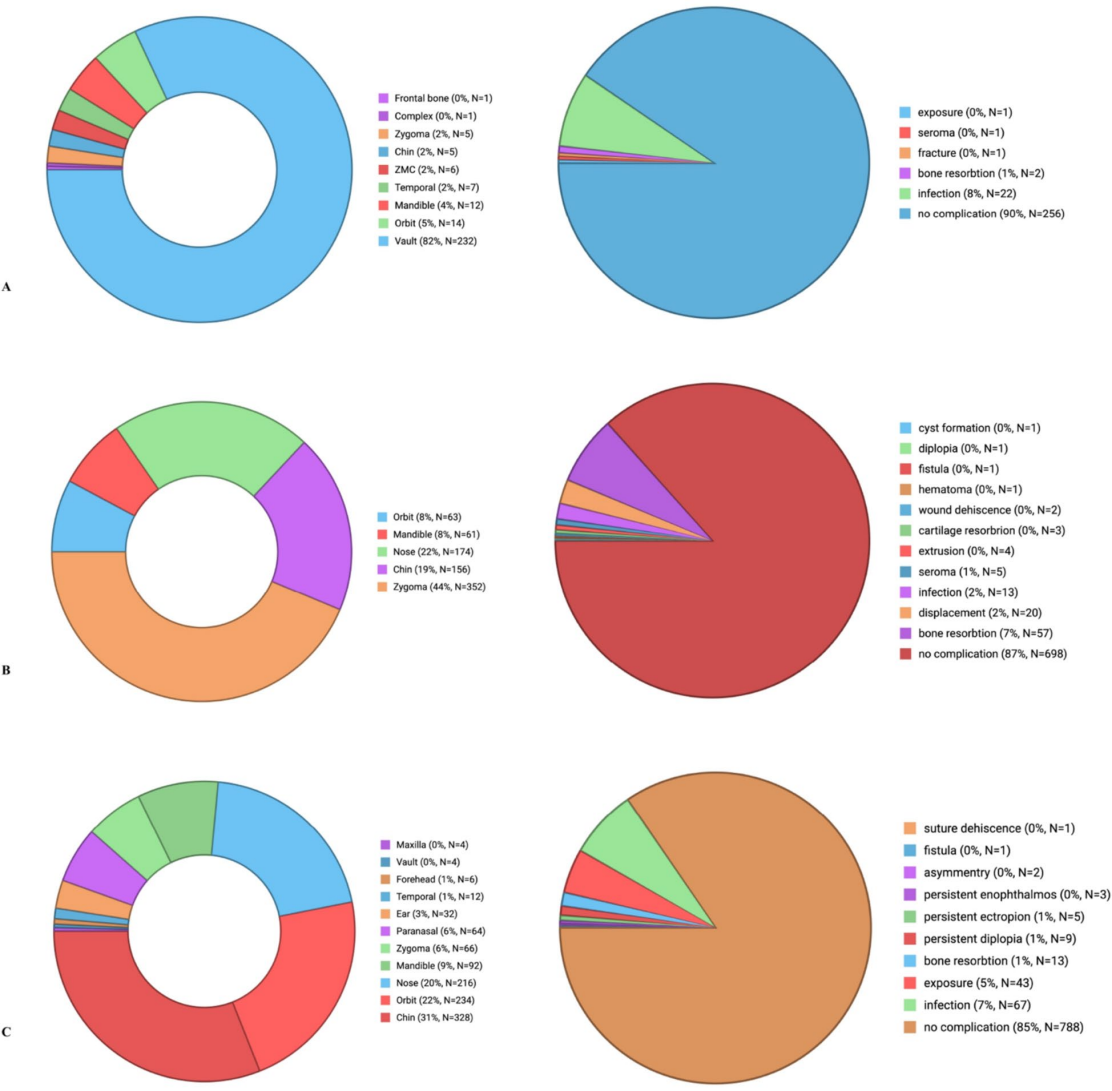

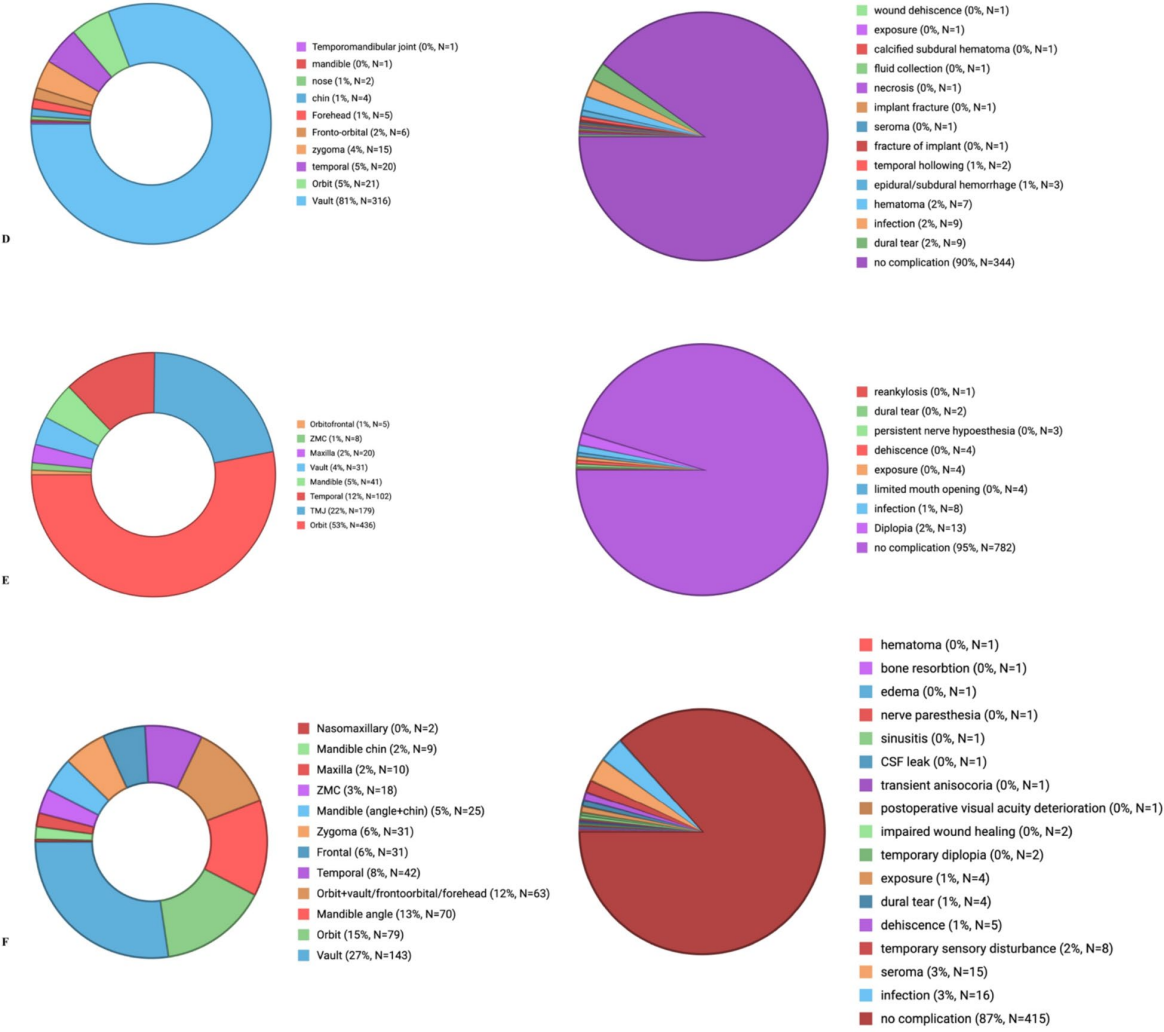
Breakdown of research sample by facial implant location and complications in** A** HA, **B** silicone, **C** polyethylene, **D** PMMA, **E** titanium, **F** PEEK implant materials. Created with BioRender.com

Anatomical areas of implementation and complications associated with different implant materials in craniomaxillofacial reconstruction were analysed and presented in Appendix B and Fig. 3. Among 3685 reconstruction cases reviewed, complication rates varied significantly across implant materials. PEEK demonstrated a complication rate of 13.4% (64/479) with the highest patient satisfaction. Titanium had a complication rate of 4.8% (39/821) and remained the preferred material for load-bearing regions, although it presented risks of exposure. PMMA showed an 11.04% (38/382) complication rate and proved effectiveness in cranial reconstructions but was associated with hematomas and dural tears. Polyethylene had the highest complication rate at 15.75% (144/914), with frequent infections and implant exposures. Silicone showed a 13.4% (108/806) complication rate, most commonly involving bone resorption and displacement. HA had a moderate complication rate of 9.5% (27/283), with infection being the primary issue.

Statistical comparison using Pearson’s Chi-square test revealed a significant difference in complication rates across the six materials (p < 0.01), underscoring the need for individualised implant selection. These findings highlight the importance of choosing implant materials based on anatomical location, functional demands, and complication profiles to optimise reconstructive outcomes and enhance patient satisfaction.

### Retrospective analysis

#### Patient characteristics

The study population comprised 22 females and 30 males with a mean age of 37.46 ± 1.36 years and with an age range of 18–67 years. The patient characteristics are presented in Table 2.

**Table 2:** Patient demographics and case characteristics

Sex	
Female	22 (42.3%)
Male	30 (57.7%)
**Etiology**	
Congenital	12 (23.1%)
Infection	2 (3.8%)
Systematic disease	2 (3.8%)
Trauma	33 (63.5%)
Tumor	3 (5.8%)
**Type of operation**	
Delayed	13 (25.0%)
Primary	39 (75.0%)
**Implant material**	
PEEK	9 (17.3%)
Silicone	14 (26.9%)
PMMA	6 (11.5%)
Titanium	23 (44.23%)
**Complications**	
Asymmetry	1 (1.9%)
Diplopia	1 (1.9%)
Exposure	2 (3.8%)
Extrusion	2 (3.8%)
Hematoma	4 (7.7%)
Infection, rejection	2 (3.8%)
Mean duration of operation in min	121.06 ± 10.306
Mean number of in-patient department bed days	6.13 ± 0.231

#### Complications

In a retrospective analysis of 52 craniomaxillofacial reconstruction cases, complications varied across the implant materials used. PEEK implants demonstrated a low complication rate, with hematoma occurring in 2 cases (22.2%) and no reports of extrusion, infection or rejection. PMMA implants showed one case of extrusion (16.7%) but no other complications. Silicone implants exhibited a broader range of complications, including asymmetry (7.1%), extrusion (7.1%) and hematoma (7.1%), accounting for a total complication rate of 21.4%. Titanium implants had the highest complication rate, with cases of asymmetry and diplopia (4.5%), exposure (9.1%), hematoma (4.5%) and infection with rejection (9.1%), resulting in a total complication rate of 22.7% (Table 3). Although PEEK demonstrated a complication rate of 22.2%, this represents only two cases of hematoma among nine patients. Similarly, complication percentages in other small subgroups (e.g. PMMA) should be interpreted with caution, as small group sizes inflate percentage values and may not reflect generalizable risk estimates.

**Table 3:** Complications across the implant materials used during craniomaxillofacial surgeries

Material of implants	Complication	Valid percent	Cumulative percent
PEEK	Hematoma	2	22.2
	No	7	77.8
PMMA	Extrusion	1	16.7
	No	5	83.3
Silicone	Asymmetry	1	7.1
	Extrusion	1	7.1
	Hematoma	1	7.1
	No	11	78.6
Titanium	Asymmetry, diplopia	1	4.5
	Exposure	2	9.1
	Hematoma	1	4.5
	Infection, rejection	2	9.1
	No	17	77.3

The initial post-hoc power analysis, based on an assumed effect size of f = 0.35, indicated that the available sample size (*n* = 52) was sufficient to detect medium-to-large differences in complication rates among the implant groups at 80% power and α = 0.05. However, the actual observed complication rates were similar across materials—22.2% for PEEK, 22.7% for titanium, and comparable frequencies for PMMA and silicone—indicating that the true effect size may be smaller than anticipated.

Given these findings, additional power calculations were conducted using more conservative effect sizes. When assuming f = 0.25 or lower, the current sample size was found to be underpowered to detect small-to-moderate differences. This limitation suggests a reduced likelihood of identifying statistically relevant variations exist, particularly given the uneven distribution of cases across the four material groups.

Therefore, non-significant results in the comparison of complication rates should be interpreted with caution. The possibility of a type II error due to insufficient power especially in detecting subtle differences cannot be excluded. These constraints highlight the need for larger, more evenly distributed samples in future research to confirm or refute observed trends.

### Aesthetic outcomes

Patient satisfaction with craniofacial reconstruction was assessed through an online survey covering five key aspects: aesthetic outcomes, symmetry, surgical site appearance, implant integration and overall recommendation. The responses revealed significant differences between the four materials studied—PEEK, PMMA, silicone and titanium which are shown in Appendix C.

For aesthetic outcomes, PEEK implants achieved the highest ratings, with 66.7% of patients marking their satisfaction as “Highly satisfied” and 33.3% as “Harmonic”, indicating a consistently positive response. PMMA had mixed results, with 16.7% of patients “Highly satisfied”, 16.7% “Harmonic” and 50% “As requested”. Silicone showed more variable results, with 42.9% rating as “Satisfied” or “As requested”, but only 7.1% achieving “Harmonic”. Titanium exhibited a wider range, with 43.5% marking “Harmonic” and 17.4% “Highly satisfied”, but also 17.4% rating as “Unsatisfied”, reflecting some dissatisfaction with its aesthetic outcomes.

For symmetry, PEEK implants again led with 55.6% of patients rating “Highly satisfied”, 33.3% “Harmonic” and 11.1% “Perfect”, showcasing excellent outcomes in this category. PMMA implants showed 66.7% “Harmonic”, 16.7% “Highly satisfied” and 16.7% “Agreed”, indicating moderate success. Silicone results were split, with 42.9% rating as “Satisfied”, 28.6% “Harmonic” and 14.3% “As requested”. Titanium showed strong results, with 39.1% “Highly satisfied”, 30.4% “Harmonic”, but also 8.7% “Unsatisfied”, reflecting variability in outcomes.

Regarding surgical site appearance, including scar visibility and contour, PEEK performed well, with 55.6% “Highly satisfied”, 33.3% “Harmonic” and 11.1% “Perfect”. PMMA implants were also favourable, with 66.7% “Harmonic”, 16.7% “Highly satisfied” and 16.7% “Satisfied”. Silicone was moderately rated, with 28.6% each for “Harmonic” and “As requested” and 21.4% “Highly satisfied” or “Satisfied”. Titanium implants had 34.8% “Highly satisfied”, 21.7% “Harmonic”, 13% “Perfect”, but 4.3% “Unsatisfied”.

**Fig. 4:**
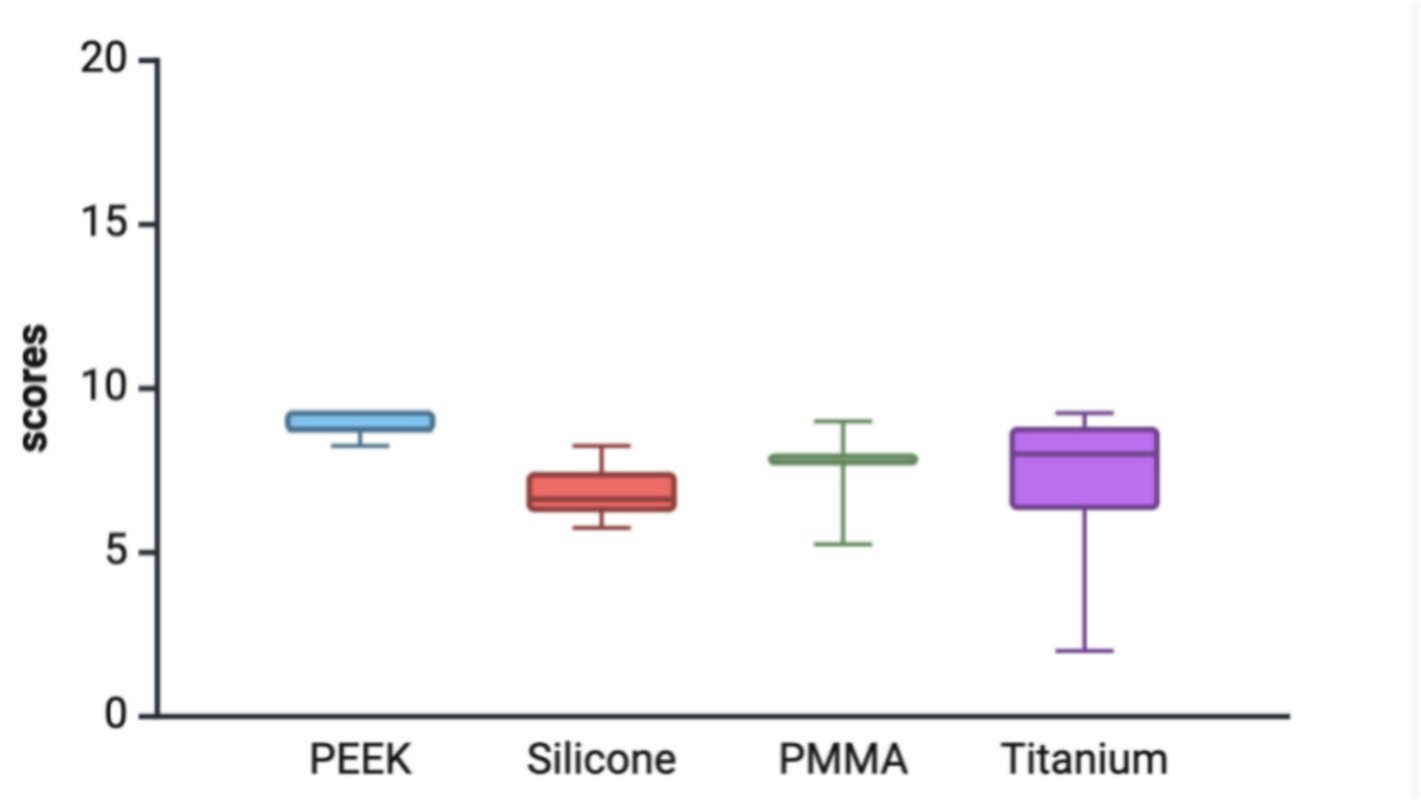
ANA scores of patient satisfaction with craniofacial reconstruction (presented as mean, minimum and maximum values). Created with BioRender.com

For implant integration with natural facial structure, PEEK achieved exceptional results, with 77.8% “Highly satisfied” and 22.2% “Perfect”, indicating complete patient approval. PMMA showed 66.7% “Harmonic”, 16.7% “Highly satisfied” and 16.7% “Agreed”. Silicone had mixed feedback, with 42.9% rating as “Satisfied” and “As requested” and 14.3% as “Harmonic”. Titanium was more varied, with 39.1% “Highly satisfied”, 21.7% “Harmonic” and 8.7% “Unsatisfied”. Average ANA scores of patient satisfaction with craniofacial reconstruction presented in Fig. 4.

Finally, for recommendations, 100% of PEEK patients would recommend the material, compared to 83.3% for PMMA, 71.4% for silicone and 73.9% for titanium.

In summary, PEEK consistently outperformed the other materials across all categories, achieving the highest satisfaction ratings for aesthetics, symmetry, site appearance and implant integration, coupled with universal patient recommendations. PEEK implants resulted in a mean ANA score of 8.86 (SD = 0.35; 8.25–9.25). Furthermore, the Kruskal–Wallis test revealed a statistically significant difference (H = 16.7605, df = 3, *p* = 0.000792).

## Discussion

The presented study examined several commonly used implant materials—PEEK, titanium, PMMA and silicone in craniomaxillofacial reconstruction. This discussion synthesizes the findings with complications reported in other studies, providing a comparative perspective.

The gathered data demonstrated the advantages of PEEK implants, including superior mechanical properties, aesthetic outcomes and patient satisfaction. In our cohort of 52 cases, PEEK showed minimal complications, with only two hematoma cases. Patient satisfaction was high, with 77.8% rating implant integration as “highly satisfied” and 100% recommending it. Comparatively, Ha et al. [81] reported complications in 17.2% of their 29 cases with PEEK and titanium implants alloy, including screw loosening, dissatisfaction and postoperative infections. Nguyen et al. (2018) documented a 4.4% infection rate in PEEK reconstructions among 136 cases. Li et al. [7] documented implant exposure in one patient (16.7%) among six patients after 10 months, while Ahmad et al. [9] observed implant removal due to recurrent infection in one case (10%) out of 10 patients. Anabtawi et al. (2021) identified complications in three cases (30%) among 10 patients, including recurrent oedema, bilateral mental nerve paraesthesia, and recurrent sinusitis. Alonso-Rodriguez et al. (2015) reported infections in two cases (14.3%) among 14 patients, with one requiring implant removal. Similarly, Kim et al. (2009) noted dehiscence in one patient (25%) among four with complex defects, and O’Reilly et al. (2015) described complications among 19 patients, including two cases of MRSA wound infections, one case of traumatic exposure, one poor cosmetic outcome and one case of epidural fluid collection. Despite these reported complications, the rates remain relatively low and are often case-specific. These findings reinforce PEEK’s reputation as a reliable material with consistent clinical and aesthetic outcomes. However, the bio-inert nature of PEEK and occasional postoperative challenges (e.g. infection or exposure) highlight the need for further innovations in surface modifications to enhance osteoconductivity and reduce infection risks.

Titanium implants had a complication rate of 22.7%, including infection, hematoma and implant exposure. Patient satisfaction was varied, with 39.1% highly satisfied but 8.7% unsatisfied. Düzgün et al. (2020) observed similar trends in their 62 orbital reconstruction cases, reporting complications such as infection (1.6%), permanent diplopia (1.6%) and enophthalmos (1.6%). Chen et al. (2015) highlighted delayed healing in 28.6% of cases, particularly in diabetic patients. Nguyen et al. (2018) reported one infection and one case of dehiscence in titanium implants. These data underscore titanium’s utility in load bearing but its limitations in aesthetics outcomes.

On the other hand, PMMA implants achieved a moderate satisfaction rate (66.7% rated symmetry as “harmonic”) but faced complications in 16.7% of cases, including implant extrusion. These align with Schön et al. (2021), who reported a 43.8% complication rate in 16 skull defect cases, including extra-axial hematomas (37.5%). Ming-Chi Hsieh et al. (2020) documented a 13.3% complication rate in occipital augmentation with PMMA implants, and Desai et al. (2019) noted a 13.3% infection rate in 30 cranioplasty cases. Our results align with these studies, highlighting its limitations in load-bearing regions and susceptibility to infection and extrusion.

Finally, silicone had a complication rate of 21.4%, including asymmetry, extrusion and patient dissatisfaction (rated appearance as “harmonic”). Al-Jandan et al. [41] reported a 4.3% infection rate and a 13.8% displacement rate in 58 mandibular augmentation cases. Similarly, Kook et al. [43] and Pelle-Ceravolo et al. [45] identified frequent complications, including infections, resorption and implant displacement. These findings mirror the results from this study, confirming the high rates of revision and mixed aesthetic outcomes associated with silicone.

PEEK demonstrates promising outcomes as an implant material, particularly in aesthetics and patient satisfaction. However, further comparative studies with longer follow-up are warranted to confirm its superiority over other materials. Despite promising findings, the claim of PEEK’s superiority must be interpreted with caution due to higher cost, limited global availability and lack of long-term multicentric studies.

Complications associated with PEEK implants appear to vary depending on the anatomical site of reconstruction. In current study, the two cases of hematoma associated with PEEK occurred in the frontal and parietal regions, where extensive subgaleal dissection and vascular plexus manipulation increase bleeding risk postoperatively. No complications were recorded in PEEK reconstruction involving the mandibular contour, zygoma or infraorbital regions, which may reflect lower vascular density and more rigid tissue compartments. This suggests that complication profiles of PEEK implants are highly site-specific, emphasising the need for tailored surgical planning based on regional anatomy, soft tissue envelope and vascularity. Optimising implant design to anatomical contours and ensuring adequate soft tissue coverage are critical to minimising complications in high-risks zones.

In conclusion, while each material has specific advantages and limitations, PEEK offers the most balanced combination of mechanical properties, aesthetic outcomes and patient satisfaction, with a lower risk of complications. Titanium remains effective for load-bearing applications but may require adjunctive measures to address aesthetic concerns. PMMA and silicone are best suited for selective cases but require caution due to their associated risks. These findings highlight the importance of personalised material selection in optimising reconstructive outcomes.

## Limitations

This study has several limitations. First, its retrospective design introduces inherent biases related to data completeness and selection. Second, the distribution of implant materials was uneven, with smaller subgroups (e.g., PEEK and PMMA) limiting the reliability of percentage-based complication estimates. Third, reliance on patient-reported surveys for aesthetic outcomes introduces subjective variability, although the structured ANA scale was applied to mitigate this. Finally, the single-center design restricts the generalizability of findings. Future studies with larger, multicentric and prospectively collected data are needed to validate these observations.

## Conclusion

This study highlights the importance of material selection in craniomaxillofacial reconstruction to achieve optimal functional and aesthetic outcomes. PEEK emerged as the most promising material due to its superior patient satisfaction, low complication rates, and balanced mechanical and aesthetic properties. However, based on the findings and literature review, implant material selection in craniomaxillofacial reconstruction should be guided by anatomical location, functional load requirements, soft tissue coverage, patient comorbidities, and aesthetic expectations. PEEK implants are most suitable for moderate-load, aesthetically sensitive regions such as the zygoma, infraorbital rim, frontal bone, and mandibular angle, where its radiolucency and contouring precision are advantageous. Titanium remains effective for high-load-bearing areas, such as mandibular body and condyle, due to its strength and osseointegration capacity. Its use in aesthetic zones may be limited when soft tissue is thin, due to increased risk of exposure and palpability. PMMA is well-suited for cranial vault and frontal bone reconstruction, especially when economic constraints or availability limit other options. However, due to its exothermic curing and infection risk, it should be avoided in load-bearing areas or where soft tissue is limited. Silicone implants are appropriate for selective aesthetic augmentations, such as the chin and nasal dorsum, particularly when cost-effective options are needed, and soft tissue coverage is sufficient. Its use should be limited in areas with dynamic soft tissue movement or bone resorption risk. Incorporating these practical considerations into preoperative planning may optimise outcomes, minimise complications and support patient-centered care.

## Supplementary Information

Supplementary Material 1.

Supplementary Material 2.

Supplementary Material 3.

## Data Availability

The datasets generated and analysed during the current study are availbale from the corresponding author upon reasonable request. Data sharing is subject to institutional and ethical regulations to protect participant confidentiality.

## Abbreviations

PEEK: Polyetheretherketone
PMMA: Polymethylmethacrylate
HA: Hydroxyapatite
PICO: The Population, Intervention, Comparison, Outcome
PRISMA: Preferred Items for Systematic Reviews and Meta-Analyses
CAD/CAM: Computer-aided design and manufacturing
3D: Three-dimensional
CT: Computed tomography
JBI: Joanna Briggs Institute
ROBINS-I: Risk of Bias in Non-Randomised Studies of Interventions
UK: The United Kingdom
NSAID: Nonsteroidal anti-inflammatory drug
MRSA: Methicillin-resistant Staphylococcus aureus
ANA: Aesthetic Numeric Analogue
