# Supplementary material for: Polyetheretherketone for craniomaxillofacial defects: cases report, evaluation of patients’ satisfaction and a systematic literature review
Source: Maxillofac Plast Reconstr Surg. 2025 Oct 24;47(1):36. doi: 10.1186/s40902-025-00482-9 (PMC12552202; doi:10.1186/s40902-025-00482-9)

**Appendix A**

**PICOS framework**

• Population (P): Patients with maxillofacial defects underwent reconstructive surgery.

• Intervention (I): Use of PEEK implants for reconstruction.

• Comparison (C): Other implant materials such as titanium, PMMA, silicone, polyethylene, hydroxyapatite.

• Outcome (O): clinical outcomes (implant succes rates, complacation rates), patient satisfaction, functional outcomes (improvement in chewing, speech, and overall quality of life).

**Research Question:**

Table 1. Eligibility criteria

|  | **Inclusion criteria** | **Exclusion criteria** |
| --- | --- | --- |
| *Patient, Population, or Problem* | Adults (≥18 years) underwent maxillofacial reconstruction using PEEK or other implants | Patents (<18 years) underwent surgery using non-maxillofacial implants (e.g., orthopedic, spinal) |
| *Intervention* | PEEK implants for craniomaxillofacial reconstruction. | PEEK used in non-reconstructive applications (e.g., dental implants, orthopedic use). |
| *Comparison Intervention/control** | Studies where other materials (titanium, PMMA, silicone, polyethylene, hydroxyapatite) were used |  |
| *Outcome* | Cinical (complications, infection rate, rejection, implant failure, symmentry)  Aesthetic (patient-reported satisfaction, aesthetic scores)  Functional (speech, chewing, quality of life improvement) | Studies not reporting clinical, aesthetic or functional oiutcomes. |
| *Study type* | Randomized controlled trials, cohort studies, case-control studies, case series. | Systematic reviews, literature reviews, animal studies, in-vitro studies. |
| *Time frame* | Published between 2000-2025 | Studies before 2000 |
| *Language* | English-language articles | Non-English articles |

**PICOS Database Search Strategy**

(“PEEK implants” OR “polyetheretherketone implants” OR “titanium implants” OR “PMMA implants” OR “silicone implants” OR “polyethylene implants” OR “hydroxyapatite implants”) AND (“maxillofacial reconstruction” OR “facial bone defects” OR “craniofacial reconstruction”) AND (“patient satisfaction” OR “clinical outcomes” OR “aesthetical outcomes” OR “complication” OR “functional outcomes”).

Table 2. PICO(S) Search Strategy

| Concept 1: | Concept 2: | *Concept 3:* | Concept 4: |
| --- | --- | --- | --- |
| maxillofacial reconstruction OR facial bone defects OR craniofacial reconstruction | PEEK implants OR  polyetheretherketone implants | titanium implants OR PMMA implants OR silicone implants OR polyethylene implants OR hydroxyapatite implants | patient satisfaction OR clinical otcomes OR aesthetical outcomes OR complication OR functional oucomes |

Table 3.MeSH Terms

| ***Keywords:*** | ***MeSH terms /Medline/PubMed:*** |
| --- | --- |
| PEEK implants | Polyetherertherketone |
| Titanium implants | Titanium |
| Polymethylmatacrylate implants | Polymethylmatacrylate |
| Silicone implants | Silicone |
| Polyethylene implants | Polyethylene |
| Hydroxyapatite implants | Hydroxyapatite |
| Cranio-, maxillofacial surgery | Maxillofacial surgery, Craniofacial Abnormalities |
| Clinical outcome | Patient Satisfaction, Treatment Outcome, Complications |

Table 4. Search Strategy

| **Electronic Database** | **Search Strategy Used** | **Filter/**  **Results** |
| --- | --- | --- |
| **MEDLINE**  **via OVID** | Ovid MEDLINE(R) ALL <1946 to 2025 May 30>  *1 Polyetheretherketone implants.mp. 53*  *2 PEEK.mp. 4193*  *3 exp Titanium/ or titanium implants.mp. 51776*  *4 exp Methylmethacrylates/ or exp Polymethyl Methacrylate/ or PMMA implants.mp. 15330*  *5 exp "Prostheses and Implants"/ or exp Silicones/ or silicone implants.mp. 643570*  *6 exp Polyethylene/ or polyethylene implants.mp. or exp Polyethylenes/ 22457*  *7 exp Hydroxyapatites/ or hydroxyapatite implants.mp. 23930*  *8 exp Plastic Surgery Procedures/ or maxillofacial reconstruction.mp. or exp Mandibular Reconstruction/ 274080*  *9 facial bone defects.mp. 25*  *10 craniofacial reconstruction.mp. 547*  *11 exp Patient Satisfaction/ or patient satisfaction.mp. 137214*  *12 exp Treatment Outcome/ or clinical outcomes.mp. 1490010*  *13 aesthetical outcomes.mp. 18*  *14 complication.mp. 400714*  *15 functional outcomes.mp. 41314*  *16 1 or 2 or 3 or 4 or 5 or 6 or 7 721421*  *17 8 or 9 or 10 274458*  *18 16 and 17 54867*  *19 11 or 12 or 13 or 14 or 15 1935967*  *20 18 and 19 21203*  *21 20 and 2020:2025.(sa_year). 5382*  *22 cranio*.mp. or exp Skull/ 324991*  *23 exp Maxillofacial Injuries/ or maxillofacial.mp. 50453*  *24 22 or 23 357433*  *25 21 and 24 836*  *Searches were updated to May 31, 2025* | **Language:** no filter  **Publication date:** 2000  onwards  **Hits: 836** |
| **Embase**  **via OVID** | Embase <1974 to 2025 May 30>  1 Polyetheretherketone implants.mp. 58  2 PEEK.mp. 5192  3 exp Titanium/ or titanium implants.mp. 65423  4 exp Methylmethacrylates/ or exp Polymethyl Methacrylate/ or PMMA implants.mp. 29454  5 exp "Prostheses and Implants"/ or exp Silicones/ or silicone implants.mp. 650340  6 exp Polyethylene/ or polyethylene implants.mp. or exp Polyethylenes/ 30362  7 exp Hydroxyapatites/ or hydroxyapatite implants.mp. 38231  8 exp Plastic Surgery Procedures/ or maxillofacial reconstruction.mp. or exp Mandibular Reconstruction/ 357701  9 facial bone defects.mp. 31  10 craniofacial reconstruction.mp. 657  11 exp Patient Satisfaction/ or patient satisfaction.mp. 208040  12 exp Treatment Outcome/ or clinical outcomes.mp. 3001233  13 aesthetical outcomes.mp. 21  14 complication.mp. 3025357  15 functional outcomes.mp. 59081  16 1 or 2 or 3 or 4 or 5 or 6 or 7 777012  17 8 or 9 or 10 357900  18 16 and 17 33370  19 11 or 12 or 13 or 14 or 15 5564474  20 18 and 19 16989  21 20 and 2020:2025.(sa_year). 5925  22 cranio*.mp. or exp Skull/ 400419  23 exp Maxillofacial Injuries/ or maxillofacial.mp. 126879  24 22 or 23 502380  25 21 and 24 716  *Searches were updated to May 31, 2025* | **Language:** no filter  **Publication date:** 2000 onwards  **Hits: 716** |
| **Cochrane Library, including Central Register of Controlled Trials (CENTRAL)** | *ID Search Hits*  *#1 polyetheretherketone OR PEEK 672*  *#2 titanium 3386*  *#3 PMMA OR Polymethylmatacrylate 587*  *#4 Polyethylene 6701*  *#5 Silicone 3525*  *#6 Hydroxyapatite 1240*  *#7 Maxillofacial surgery 5195*  *#8 Patient satisfaction OR Complication 143870*  *#9 (Polyetherertherketone OR Titanium OR Polymethylmatacrylate OR Silicone OR Polyethylene OR Hydroxyapatite):ti,ab,kw AND (Maxillofacial surgery OR facial bone defects OR craniofacial):ti,ab,kw (Word variations have been searched) 227*  *Searches were updated to May 31, 2025* | **Language:** no filter  **Publication date:** 2000 onwards  **Hits: 227** |

Table 6. Included Studies

| **№** | **Study** |
| --- | --- |
|  | Ahmad AF, Yaakob H, Khalil A, Georges P. Evaluating patients' satisfaction level after using 3D printed PEEK facial implants in repairing maxillofacial deformities. Ann Med Surg (Lond). 2022;79:104095. |
|  | Al-Jandan B, Marei HF. Mandibular angle augmentation using solid silicone implants. Dent Med Probl. 2018;55(4):367-70. |
|  | Al-Sukhun J. A Novel Method to Reconstruct the Upper and Lower Jaws Using 3D-Custom-Made Titanium Implants. J Craniofac Surg. 2023;34(3):e244-e6. |
|  | Alasseri N, Alasraj A. Patient-specific implants for maxillofacial defects: challenges and solutions. Maxillofac Plast Reconstr Surg. 2020;42(1):15. |
|  | Alonso-Rodriguez E, Cebrián JL, Nieto MJ, Del Castillo JL, Hernández-Godoy J, Burgueño M. Polyetheretherketone custom-made implants for craniofacial defects: Report of 14 cases and review of the literature. Journal of Cranio-Maxillofacial Surgery. 2015;43(7):1232-8. |
|  | Anabtawi M, Thomas M, Lee NJ. The Use of Interlocking Polyetheretherketone (PEEK) Patient-Specific Facial Implants in the Treatment of Facial Deformities. A Retrospective Review of Ten Patients. J Oral Maxillofac Surg. 2021;79(5):1145.e1-.e9. |
|  | Atef M, Mounir M, Shawky M, Mounir S, Gibaly A. Polyetheretherketone patient-specific implants (PPSI) for the reconstruction of two different mandibular contour deformities. Oral Maxxillofac Surg. 2022;26(2):299-309. |
|  | Bai SS, Li D, Xu L, Duan HC, Yuan J, Wei M. A Novel Method to Enhance Dynamic Rhinoplasty Outcomes: Double "V" Carving for Alloplastic Grafts. Ear Nose Throat J. 2020;99(4):262-7. |
|  | Bassi M, Antonelli V, Tomassini A, Maimone G, D'Andrea M, Campobassi A, et al. Synchronized "One-Step" Resection and Cranio-Orbital Reconstruction for Spheno-Orbital Lesions With Custom Made Implant. J Craniofac Surg. 2021;32(5):1870-3. |
|  | Brandicourt P, Delanoé F, Roux F-E, Jalbert F, Brauge D, Lauwers F. Reconstruction of Cranial Vault Defect with Polyetheretherketone Implants. World Neurosurgery. 2017;105:783-9. |
|  | Brie J, Chartier T, Chaput C, Delage C, Pradeau B, Caire F, et al. A new custom made bioceramic implant for the repair of large and complex craniofacial bone defects. J Craniomaxillofac Surg. 2013;41(5):403-7. |
|  | Bruens ML, Pieterman H, de Wijn JR, Vaandrager JM. Porous polymethylmethacrylate as bone substitute in the craniofacial area. J Craniofac Surg. 2003;14(1):63-8. |
|  | Carloni R, Herlin C, Chaput B, De Runz A, Watier E, Bertheuil N. Scalp Tissue Expansion Above a Custom-Made Hydroxyapatite Cranial Implant to Correct Sequelar Alopecia on a Transposition Flap. World Neurosurg. 2016;95:616.e1-.e5. |
|  | Chang PC. Computer-Assisted Planning and 3D Printing-Assisted Modeling for Chin Augmentation. Aesthet Surg J. 2017;38(1):1-10. |
|  | Chattopadhyay C. Reconstruction of Acquired Frontal Bone Defects Using Titanium Mesh Implants: A Retrospective Study. J Maxillofac Oral Surg. 2019;18(1):34-9. |
|  | Chen ST, Chang CJ, Su WC, Chang LW, Chu IH, Lin MS. 3-D titanium mesh reconstruction of defective skull after frontal craniectomy in traumatic brain injury. Injury. 2015;46(1):80-5. |
|  | Colletti G, Saibene AM, Giannini L, Dessy M, Deganello A, Pipolo C, et al.  Endoscopic endonasal repair with polyethylene implants in medial orbital wall fractures: A prospective study on 25 cases. J Craniomaxillofac Surg. 2018;46(2):274-82. |
|  | Copperman TS, Idowu OO, Jalaj S, Winn BJ, Pham C, Setabutr P, et al. Patient-Specific Implants in Oculofacial Plastic Surgery. Ophthalmic Plast Reconstr Surg. 2021;37(3):241-7. |
|  | da Silva de Menezes JD, Moura LB, Martins RP, Hochuli-Vieira E. Porous Polyethylene Implant as Aesthetic Complement in Orthognathic Surgery. J Craniofac Surg. 2016;27(8):e790-e1. |
|  | Desai JB. Cost-Effective Technique of Fabrication of Polymethyl Methacrylate Based Cranial Implant Using Three-Dimensional Printed Moulds and Wax Elimination Technique. J Craniofac Surg. 2019;30(4):1259-63. |
|  | Englar KM, Kordahi AM, Brandel MG, Santiago-Dieppa DR, Wali AR, Pham M, et al. Application of Antibiotic-Impregnated Polymethyl-Methacrylate Bone Cement for the Treatment of Infected Cranioplasties: Initial Experience. Ann Plast Surg. 2022;88(4 Suppl 4):S357-s60. |
|  | Eolchiyan SA. Complex skull defects reconstruction with САD/САМ titanium and polyetheretherketone (PEEK) implants. Zh Vopr Neirokhir Im N N Burdenko. 2014;78(4):3-13. |
|  | Gerbino G, Bianchi FA, Zavattero E, Tartara F, Garbossa D, Ducati A. Single-step resection and reconstruction using patient-specific implants in the treatment of benign cranio-orbital tumors. J Oral Maxillofac Surg. 2013;71(11):1969-82. |
|  | Gerbino G, Zavattero E, Zenga F, Bianchi FA, Garzino-Demo P, Berrone S. Primary and secondary reconstruction of complex craniofacial defects using polyetheretherketone custom-made implants. J Craniomaxillofac Surg. 2015;43(8):1356-63. |
|  | Groth MJ, Bhatnagar A, Clearihue WJ, Goldberg RA, Douglas RS. Long-term efficacy of biomodeled polymethyl methacrylate implants for orbitofacial defects. Arch Facial Plast Surg. 2006;8(6):381-9. |
|  | Guo JS, Ng KLB, Lee SS, Lai YW, Wu YC. Custom-Made Implant Fabrication for Chin Augmentation Using Piled-Up Expanded Polytetrafluoroethylene Sheets: An Innovative Surgical Technique and Literature Review. Aesthetic Plast Surg. 2024;48(11):2018-24. |
|  | Guo J, Tian W, Long J, Gong H, Duan S, Tang W. A retrospective study of traumatic temporal hollowing and treatment with titanium mesh. Ann Plast Surg. 2012;68(3):279-85. |
|  | Habib LA, Yoon MK. Patient specific implants in orbital reconstruction: A pilot study. Am J Ophthalmol Case Rep. 2021;24:101222. |
|  | Han X, Sharma N, Xu Z, Scheideler L, Geis-Gerstorfer J, Rupp F, et al. An In Vitro Study of Osteoblast Response on Fused-Filament Fabrication 3D Printed PEEK for Dental and Cranio-Maxillofacial Implants. J Clin Med. 2019;8(6). |
|  | Hatamleh MM, Bhamrah G, Ryba F, Mack G, Huppa C. Simultaneous Computer-Aided Design/Computer-Aided Manufacture Bimaxillary Orthognathic Surgery and Mandibular Reconstruction Using Selective-Laser Sintered Titanium Implant. J Craniofac Surg. 2016;27(7):1810-4. |
|  | He Q, Rao P, Wang L, Li Y, Fu G, Xiao J. Reconstruction of orbital wall fractures with superior orbital fissure syndrome using individualized preformed titanium mesh: a pilot study. Oral Surg Oral Med Oral Pathol Oral Radiol. 2023;135(1):24-32. |
|  | Helmers R, Klop C, Schreurs R, de Lange J, Dubois L. Minimally Invasive Treatment With a Patient Specific Implant in Reconstruction of Isolated Anterior Wall Fracture of the Frontal Sinus. J Craniofac Surg. 2021;32(1):341-4. |
|  | Hoang TA, Lee KC, Dung V, Chuang SK. Augmentation Rhinoplasty in Cleft Lip Nasal Deformity Using Alloplastic Material and Autologous Cartilage. J Craniofac Surg. 2022;33(8):e883-e6. |
|  | Huang Q, Yang B, Li B, Ma H. A New Type of Three-Dimensional Customized Composite Implant in Reconstruction of Large Skull Defects. J Craniofac Surg. 2021;32(3):1045-8. |
|  | Huang GJ, Zhong S, Susarla SM, Swanson EW, Huang J, Gordon CR. Craniofacial reconstruction with poly(methyl methacrylate) customized cranial implants. J Craniofac Surg. 2015;26(1):64-70. |
|  | Hwang J, Park B. Correction of the Deepened Labiomental Groove Using Silicone Implants in Advancement Genioplasty. J Craniofac Surg. 2021;32(4):e389-e92. |
|  | Iaccarino C, Viaroli E, Fricia M, Serchi E, Poli T, Servadei F. Preliminary Results of a Prospective Study on Methods of Cranial Reconstruction. J Oral Maxillofac Surg. 2015;73(12):2375-8. |
|  | Jain R, Mahendru S, Aggarwal A, Brajesh V, Aulakh HS, Singh S, et al. Feasibility of Customised Polymethyl Methacrylate Implants Fabricated Using 3D Printed Flexible Moulds for Correction of Facial Skeletal Deformities. J Craniofac Surg. 2021;32(6):1981-5. |
|  | Jalbert F, Boetto S, Nadon F, Lauwers F, Schmidt E, Lopez R. One-step primary reconstruction for complex craniofacial resection with PEEK custom-made implants. J Craniomaxillofac Surg. 2014;42(2):141-8. |
|  | Jiang C, Zhao C, Chen B, Lu L, Sun Y, Yan X, et al. Auricular reconstruction using Medpor combined with different hearing rehabilitation approaches for microtia. Acta Otolaryngol. 2021;141(6):572-8. |
|  | Kärkkäinen M, Wilkman T, Mesimäki K, Snäll J. Primary reconstruction of orbital fractures using patient-specific titanium milled implants: the Helsinki protocol. Br J Oral Maxillofac Surg. 2018;56(9):791-6. |
|  | Kattimani VS, Chakravarthi PS, Prasad LK. Biograft Block Hydroxyapatite: A Ray of Hope in the Reconstruction of Maxillofacial Defects. J Craniofac Surg. 2016;27(1):247-52. |
|  | Kim MM, Boahene KD, Byrne PJ. Use of customized polyetheretherketone (PEEK) implants in the reconstruction of complex maxillofacial defects. Arch Facial Plast Surg. 2009;11(1):53-7. |
|  | Kook WS, Ryu DH, Baek W, Kook HM, Jang YY, Lew DH. Prevention and Resolution of Silicone Implant-Related Problems in Secondary Rhinoplasty Using a Cross-Linked Human Acellular Dermal Matrix. Plast Reconstr Surg. 2023;152(1):45-54. |
|  | Landry M, Hankins M, Berkovic J, Nathan CA. Delayed Infection of Porous Polyethylene Implants After Oncologic Maxillectomy and Reconstruction: 2 Case Reports and Review of Literature. Ear Nose Throat J. 2021;100(10_suppl):1023s-6s. |
|  | Lee UL, Kwon JS, Woo SH, Choi YJ. Simultaneous Bimaxillary Surgery and Mandibular Reconstruction With a 3-Dimensional Printed Titanium Implant Fabricated by Electron Beam Melting: A Preliminary Mechanical Testing of the Printed Mandible. J Oral Maxillofac Surg. 2016;74(7):1501.e1-.e15. |
|  | Leiser Y, Shilo D, Wolff A, Rachmiel A. Functional Reconstruction in Mandibular Avulsion Injuries. J Craniofac Surg. 2016;27(8):2113-6. |
|  | Li Y, Li Z, Tian L, Li D, Lu B, Shi C, et al. Clinical application of 3D-printed PEEK implants for repairing mandibular defects. J Craniomaxillofac Surg. 2022;50(8):621-6. |
|  | Liebelt BD, Huang M, Baskin DS. Sellar Floor Reconstruction with the Medpor Implant Versus Autologous Bone After Transnasal Transsphenoidal Surgery: Outcome in 200 Consecutive Patients. World Neurosurg. 2015;84(2):240-5. |
|  | Li P, Shen L, Li J, Liang R, Tian W, Tang W. Optimal design of an individual endoprosthesis for the reconstruction of extensive mandibular defects with finite element analysis. J Craniomaxillofac Surg. 2014;42(1):73-8. |
|  | Liu Y, Xie S, Ding J, Zhang Y, Deng L, Yao Y, et al. Complex Frontal Bone Reconstruction Using Computer-designed Polyetheretherketone Implant: Case Report and Literature Review. Plast Reconstr Surg Glob Open. 2024;12(8):e6007. |
|  | Long J, Zhang J, Kang J, Fan Y, Zhang Z, Shi J, et al. Customed 3D-printed Polyetheretherketone (PEEK) Implant for Secondary Salvage Reconstruction of Mandibular Defects: Case Report and Literature Review. J Craniofac Surg. 2023;34(8):2460-3. |
|  | Lutz JC, Assouline Vitale LS, Graillon N, Foletti JM, Schouman T. Standard and Customized Alloplastic Facial Implants Refining Orthognathic Surgery: Outcome Evaluation. J Oral Maxillofac Surg. 2020;78(10):1832.e1-.e12. |
|  | Maissen M, Gander T. Retrospective comparison of one-stage and two-stage orbital reconstruction in patients suffering from combined injuries of the midface. Br J Oral Maxillofac Surg. 2023;61(4):289-94. |
|  | Martinez-Seijas P, Díaz-Galvis LA, Hernando J, Leizaola-Cardesa IO, Aguilar-Salvatierra A, Gómez-Moreno G. Polymethyl Methacrylate Custom-Made Prosthesis: A Novel Three-Dimension Printing-Aided Fabrication Technique for Cranial and/or Orbital Reconstruction. J Craniofac Surg. 2018;29(5):e438-e40. |
|  | Melville JC, Manis CS, Shum JW, Alsuwied D. Single-Unit 3D-Printed Titanium Reconstruction Plate for Maxillary Reconstruction: The Evolution of Surgical Reconstruction for Maxillary Defects-A Case Report and Review of Current Techniques. J Oral Maxillofac Surg. 2019;77(4):874.e1-.e13. |
|  | Milhomem AC, Alves LM, de Souza Jorge IM, Costa EL, Vinaud MC, de Souza Lino Júnior R. Facial trauma reconstruction with polymethyl methacrylate-A case report. J Cosmet Dermatol. 2018;17(6):1037-40. |
|  | Ming-Chi Hsieh A, Kuan-Chou Lin C, Hou-Ren Jiang S, Seah TE. Esthetic Occipital Augmentation With Computer-Aided Design-Computer-Aided Manufacturing Prefabricated Customized Polymethyl Methacrylate Implant: Comparison of Planned and Final Results. J Oral Maxillofac Surg. 2020;78(7):1191.e1-.e8. |
|  | Marchac D, Greensmith A. Long-term experience with methylmethacrylate cranioplasty in craniofacial surgery. J Plast Reconstr Aesthet Surg. 2008;61(7):744-52; discussion 53. |
|  | Morice A, Kolb F, Picard A, Kadlub N, Puget S. Reconstruction of a large calvarial traumatic defect using a custom-made porous hydroxyapatite implant covered by a free latissimus dorsi muscle flap in an 11-year-old patient. J Neurosurg Pediatr. 2017;19(1):51-5. |
|  | Mounir M, Abou-ElFetouh A, ElBeialy W, Mounir R. Patient-specific alloplastic endoprosthesis for reconstruction of the mandible following segmental resection: A case series. J Craniomaxillofac Surg. 2020;48(8):719-23. |
|  | Narciso R, Basile E, Bottini DJ, Cervelli V. PEEK Implants: An Innovative Solution for Facial Aesthetic Surgery. Case Reports in Surgery. 2021;2021:5518433. |
|  | Nguyen PD, Khechoyan DY, Phillips JH, Forrest CR. Custom CAD/CAM implants for complex craniofacial reconstruction in children: Our experience based on 136 cases. J Plast Reconstr Aesthet Surg. 2018;71(11):1609-17. |
|  | Niechajev I. Facial reconstruction using porous high-density polyethylene (medpor): long-term results. Aesthetic Plast Surg. 2012;36(4):917-27. |
|  | Nocini R, D'Agostino A, Trevisiol L, Favero V. Mandibular recontouring with polyetheretherketone (PEEK) patient-specific implants. BMJ Case Rep. 2022;15(4). |
|  | O'Reilly EB, Barnett S, Madden C, Welch B, Mickey B, Rozen S. Computed-tomography modeled polyether ether ketone (PEEK) implants in revision cranioplasty. J Plast Reconstr Aesthet Surg. 2015;68(3):329-38. |
|  | Olate S, Uribe F, Huentequeo-Molina C, Goulart DR, Sigua-Rodriguez EA, Alister JP. Mandibular Angle Contouring Using Porous Polyethylene Stock or PEEK-based Patient Specific Implants. A Critical Analysis. J Craniofac Surg. 2021;32(1):242-6. |
|  | Pelle-Ceravolo M, Angelini M. Zygomatic Implants through a Face-Lift Approach: Analysis of 30 Years' Experience. Plast Reconstr Surg. 2022;150(5):995-1005. |
|  | Pietzka S, Wenzel M, Winter K, Wilde F, Schramm A, Ebeling M, et al. Comparison of Anatomical Preformed Titanium Implants and Patient-Specific CAD/CAM Implants in the Primary Reconstruction of Isolated Orbital Fractures-A Retrospective Study. J Pers Med. 2023;13(5). |
|  | Probst FA, Cornelius CP, Otto S, Malenova Y, Probst M, Liokatis P, et al. Accuracy of free-hand positioned patient specific implants (PSI) in primary reconstruction after inferior and/or medial orbital wall fractures. Comput Biol Med. 2021;137:104791. |
|  | Puricelli E. Puricelli biconvex arthroplasty as an alternative for temporomandibular joint reconstruction: description of the technique and long-term case report. Head Face Med. 2022;18(1):27. |
|  | Sainsbury DC, George A, Forrest CR, Phillips JH. Bilateral Malar Reconstruction Using Patient-Specific Polyether Ether Ketone Implants in Treacher-Collins Syndrome Patients With Absent Zygomas. J Craniofac Surg. 2017;28(2):515-7. |
|  | Schön SN, Skalicky N, Sharma N, Zumofen DW, Thieringer FM. 3D-Printer-Assisted Patient-Specific Polymethyl Methacrylate Cranioplasty: A Case Series of 16 Consecutive Patients. World Neurosurg. 2021;148:e356-e62. |
|  | Schwaiger M, Echlin K, Atherton D, Haers P. The use of Medpor implants for midface contouring in cleft patients. Int J Oral Maxillofac Surg. 2019;48(9):1177-84. |
|  | Scopelliti D, Amodeo G. Nasal Dorsum Reconstruction With Alloplastic Material. J Craniofac Surg. 2016;27(7):e676-e8. |
|  | Sendul SY, Yildiz AM, Yildiz AA, Akbas E. Osseointegrated Implants for Orbito-Facial Prostheses: Common Complications and Solutions. J Craniofac Surg. 2021;32(5):1770-4. |
|  | Sesqué A, Dang NP, Coste A, Barthélémy I, Depeyre A. Orbitofrontal Reconstruction With a Three-Dimensional Titanium Patient-Specific Implant After Intraosseous Haemangioma Resection. J Craniofac Surg. 2021;32(1):e69-e72.  129. |
|  | Systermans S, Cobraiville E, Camby S, Meyer C, Louvrier A, Lie SA, et al. An innovative 3D hydroxyapatite patient-specific implant for maxillofacial bone reconstruction: A case series of 13 patients. J Craniomaxillofac Surg. 2024;52(4):420-31. |
|  | Touré G, Gouet E. Use of a 3-Dimensional Custom-Made Porous Titanium Prosthesis for Mandibular Body Reconstruction With Prosthetic Dental Rehabilitation and Lipofilling. J Oral Maxillofac Surg. 2019;77(6):1305-13. |
|  | Verbist M, Vandevelde AL, Geusens J, Sun Y, Shaheen E, Willaert R. Reconstruction of Craniomaxillofacial Bone Defects with 3D-Printed Bioceramic Implants: Scoping Review and Clinical Case Series. J Clin Med. 2024;13(10). |
|  | Wei LA, Brown JJ, Hosek DK, Burkat CN. Osseointegrated implants for orbito-facial prostheses: Preoperative planning tips and intraoperative pearls. Orbit. 2016;35(2):55-61. |
|  | Wongsirisuwan M. Customized anterior temporal augmentation for treating anterior temporal hollowing (ATH) by 3D-printed cranioplasty. Neurochirurgie. 2024;70(1):101528. |
|  | Woo JM, Baek SH, Kim JC, Choi JY. Contour Restoration of Over-Resected Mandibular Angle and Lower Border by Reduction Mandibuloplasty Using Three-Dimensional Planning and Computer-Aided Design and Manufacturing Custom-Made Titanium Implants. J Craniofac Surg. 2018;29(4):e340-e3. |
|  | Yan K, Wu Y, Xie Z, Yan S, Qiao C, Qu Y, et al. Endoscopic-Assisted Forehead Augmentation with Polyetheretherketone (PEEK) Patient-Specific Implant (PSI) for Aesthetic Considerations. Aesthetic Plast Surg. 2024;48(10):1889-98. |
|  | Yim HW, Nguyen A, Kim YK. Facial Contouring Surgery with Custom Silicone Implants Based on a 3D Prototype Model and CT-Scan: A Preliminary Study. Aesthetic Plast Surg. 2015;39(3):418-24. |
|  | Zhang L, Shen S, Yu H, Shen SG, Wang X. Computer-Aided Design and Computer-Aided Manufacturing Hydroxyapatite/Epoxide Acrylate Maleic Compound Construction for Craniomaxillofacial Bone Defects. J Craniofac Surg. 2015;26(5):1477-81. |
|  | Zhang T, Young S, Lang SS, Sundar G. Prebending of Prefabricated Orbital Implants: Towards Improved Orbital Angle Symmetry Post Craniofacial Trauma Surgery. J Craniofac Surg. 2022;33(3):740-3. |
|  | Zimmerer RM, Ellis E, 3rd, Aniceto GS, Schramm A, Wagner ME, Grant MP, et al. A prospective multicenter study to compare the precision of posttraumatic internal orbital reconstruction with standard preformed and individualized orbital implants. J Craniomaxillofac Surg. 2016;44(9):1485-97. |
|  | Saponaro G, Doneddu P, Gasparini G, Staderini E, Boniello R, Todaro M, et al. Custom made onlay implants in peek in maxillofacial surgery: a volumetric study. Childs Nerv Syst. 2020;36(2):385-91. |
|  | Saponaro G, Todaro M, Barbera G, Scivoletto G, Foresta E, Gasparini G, et al. Patient-Specific Facial Implants in Polyetheretherketone and Their Stability: A Preliminary Study. Ann Plast Surg. 2023;90(6):564-7. |
|  | Khashaba M, Shawky M. Patient-specific PEEK implant for treatment of temporal myositis ossificans (five years follow-up): A case report. J Stomatol Oral Maxillofac Surg. 2023;124(6s):101593. |
|  | Shi H, Yin X, Hu Y. Solitary Neurofibroma of the Zygoma: Three-Dimensional Virtual Resection and Patient-Specific Polyetheretherketone Implant Reconstruction. J Craniofac Surg. 2022;33(8):e781-e3. |
|  | Rezai A, Pöppe JP, Gaggl A, Griessenauer CJ, Schwartz C, Krainz H, et al. Single-step 3D printing aided cranio-orbital reconstruction with patient specific polyetheretherketone implants after resection of benign spheno-orbital tumors. Acta Neurochir (Wien). 2024;166(1):499. |
|  | Pöppe JP, Spendel M, Griessenauer CJ, Gaggl A, Wurm W, Enzinger S. Point-of-Care 3-Dimensional-Printed Polyetheretherketone Customized Implants for Cranioplastic Surgery of Large Skull Defects. Oper Neurosurg (Hagerstown). 2024;27(4):449-54. |
|  | Pöppe JP, Spendel M, Schwartz C, Winkler PA, Wittig J. The "springform" technique in cranioplasty: custom made 3D-printed templates for intraoperative modelling of polymethylmethacrylate cranial implants. Acta Neurochir (Wien). 2022;164(3):679-88. |
|  | Todaro M, Saponaro G, Perquoti F, Gasparini G, Signorelli F, Tartaglione T, et al. Bone Regeneration and Polyetheretherketone Implants in Maxillo-Facial Surgery and Neurosurgery: A Multidisciplinary Study. Biology (Basel). 2024;13(7). |
|  | Ali S, Abdel Aziz O, Ahmed M. Patient-specific PEEK implants for immediate restoration of temporal fossa after maxillary reconstruction with temporalis muscle flap. Maxillofac Plast Reconstr Surg. 2022;44(1):20. |
|  | Cárdenas-Serres C, Almeida-Parra F, Simón-Flors AM, de Leyva-Moreno P, Ranz-Colio Á, Ley-Urzaiz L, et al. Custom CAD/CAM Peek Implants for Complex Orbitocranial Reconstruction: Our Experience with 15 Patients. J Clin Med. 2024;13(3). |
|  | Chepurnyi Y, Chernogorskyi D, Kopchak A, Petrenko O. Clinical efficacy of peek patient-specific implants in orbital reconstruction. J Oral Biol Craniofac Res. 2020;10(2):49-53. |
|  | Dean A, Estévez O, Centella C, Sanjuan-Sanjuan A, Sánchez-Frías ME, Alamillos FJ. Surgical Navigation and CAD-CAM-Designed PEEK Prosthesis for the Surgical Treatment of Facial Intraosseous Vascular Anomalies. J Clin Med. 2024;13(16). |
|  | Hamsho R, Mahardawi B, Assi H, Alkhatib H. Polyetheretherketone (PEEK) Implant for the Reconstruction of Severe Destruction in the Maxilla: Case Report. Plast Reconstr Surg Glob Open. 2022;10(8):e4473. |
|  | Liu BY, Cao G, Dong Z, Chen W, Xu JK, Guo T. The application of 3D-printed titanium mesh in maxillary tumor patients undergoing total maxillectomy. J Mater Sci Mater Med. 2019;30(11):125. |
|  | Gugliotta Y, Zavattero E, Ramieri G, Borbon C, Gerbino G. Cranio-Maxillo-Facial Reconstruction with Polyetheretherketone Patient-Specific Implants: Aesthetic and Functional Outcomes. J Pers Med. 2024;14(8). |
|  | van der Wel H, Merema BJ, Kraeima J, Schepers RH, Jansma J. Facial Masculinization Surgery Using Polyetheretherketone Alloplasty: Statistical Shape Modeling-based Implant Designs. Plast Reconstr Surg Glob Open. 2024;12(7):e6012. |
|  | Kerkfeld V, Schorn L, Depprich R, Lommen J, Wilkat M, Kübler N, et al. Simultaneous PSI-Based Orthognathic and PEEK Bone Augmentation Surgery Leads to Improved Symmetric Facial Appearance in Craniofacial Malformations. J Pers Med. 2022;12(10). |
|  | Yang M, Wu Z, Yu H, Cheng J. Reconstruction for diverse fronto-orbital defects with computer-assisted designed and computer-assisted manufactured PEEK implants in one-stage operation: Case reports. Medicine (Baltimore). 2021;100(40):e27452. |
|  | Yan S, Qiao C, Yan K, Qu Y, Wang S, Shangguan W, et al. Endoscopic-Assisted Mandibular Angle Revision Using Patient-Specific PEEK Implants: Surgical Accuracy and Aesthetic Outcomes in Over-Resected Mandibles. Aesthetic Plast Surg. 2025. |
|  | Komal S, Kasturi B, Paranjyoti B, Rahul G. An unusual encounter in ophthalmology: Fronto-Ethemoido-Orbital Osteoma with the art of precise referral and management of PEEK implant infection- A rare case report. Am J Ophthalmol Case Rep. 2025;37:102254. |
|  | Lv M, Yang X, Gvetadze SR, Gupta A, Li J, Sun J. Accurate reconstruction of bone defects in orbital-maxillary-zygomatic (OMZ) complex with polyetheretherketone (PEEK). J Plast Reconstr Aesthet Surg. 2022;75(5):1750-7. |
|  | Daniel E, Browne JD. Minimizing complications in the use of titanium condylar head reconstruction prostheses. Otolaryngol Head Neck Surg. 2004;130(3):344-50. |
|  | Yates JM, Wildgoose DG, van Noort R. Correction of a mandibular asymmetry using a custom-made titanium onlay. J Plast Reconstr Aesthet Surg. 2009;62(8):e247-50. |
|  | Tang W, Long J, Feng F, Guo L, Gao C, Tian W. Condyle replacement after tumor resection: comparison of individual prefabricated titanium implants and costochondral grafts. Oral Surg Oral Med Oral Pathol Oral Radiol Endod. 2009;108(2):147-52. |
|  | Mustafa SF, Evans PL, Bocca A, Patton DW, Sugar AW, Baxter PW. Customized titanium reconstruction of post-traumatic orbital wall defects: a review of 22 cases. Int J Oral Maxillofac Surg. 2011;40(12):1357-62. |
|  | Dediol E, Uglešić V, Zubčić V, Knežević P. Brown class III maxillectomy defects reconstruction with prefabricated titanium mesh and soft tissue free flap. Ann Plast Surg. 2013;71(1):63-7. |
|  | Watson J, Hatamleh M, Alwahadni A, Srinivasan D. Correction of facial and mandibular asymmetry using a computer aided design/computer aided manufacturing prefabricated titanium implant. J Craniofac Surg. 2014;25(3):1099-101. |
|  | Kanatsios S, Breik O, Dimitroulis G. Biomet stock temporomandibular joint prosthesis: Long-term outcomes of the use of titanium condyles secured with four or five condylar fixation screws. J Craniomaxillofac Surg. 2018;46(10):1697-702. |
|  | Xue R, Lai Q, Sun S, Lai L, Tang X, Ci J, et al. Application of Three-Dimensional Printing Technology for Improved Orbital-Maxillary-Zygomatic Reconstruction. J Craniofac Surg. 2019;30(2):e127-e31. |
|  | Vrielinck L, Sun Y, Schepers S, Politis C, Van Slycke S, Agbaje JO. Osseous reconstruction using an occlusive titanium membrane following marginal mandibulectomy: proof of principle. J Craniofac Surg. 2014;25(3):1112-4. |
|  | Henry A, Inverso G, Granquist EJ. Revision temporomandibular joint arthroplasty for the treatment of acquired metal allergy and review of the literature. Int J Oral Maxillofac Surg. 2020;49(3):356-60. |
|  | Cortese A, Spirito F, Claudio PP, Lo Muzio L, Ruggiero A, Gargiulo M. Mandibular Reconstruction after Resection of Ameloblastoma by Custom-Made CAD/CAM Mandibular Titanium Prosthesis: Two Case Reports, Finite Element Analysis and Discussion of the Technique. Dent J (Basel). 2023;11(4). |
|  | Xia Y, Feng ZC, Li C, Wu H, Tang C, Wang L, et al. Application of additive manufacturing in customized titanium mandibular implants for patients with oral tumors. Oncol Lett. 2020;20(4):51. |
|  | Rajkumar A, Sidebottom AJ. Prospective study of the long-term outcomes and complications after total temporomandibular joint replacement: analysis at 10 years. Int J Oral Maxillofac Surg. 2022;51(5):665-8. |
|  | Kundakçioğlu A, Bilazeroğlu E, Emes Y, Aybar B. Virtual Surgical Planning and Customized Subperiosteal Implant for Reconstruction of Maxillary Defect After Oncological Resection. J Craniofac Surg. 2024. |
|  | Chamo D, Msallem B, Sharma N, Aghlmandi S, Kunz C, Thieringer FM. Accuracy Assessment of Molded, Patient-Specific Polymethylmethacrylate Craniofacial Implants Compared to Their 3D Printed Originals. J Clin Med. 2020;9(3). |
|  | Dib J, Ferreira M, Dib M, Silva W, Neves R, Torres É, et al. Cranioplasty using Polymethylmethacrylate (PMMA) self-curing by the conventional casting process: clinical case report. RGO - Revista Gaúcha de Odontologia. 2018;66:267-73. |
|  | Giese H, Meyer J, Engel M, Unterberg A, Beynon C. Polymethylmethacrylate patient-matched implants (PMMA-PMI) for complex and revision cranioplasty: analysis of long-term complication rates and patient outcomes. Brain Inj. 2020;34(2):269-75. |
|  | Elgazzar K, Elshahat A. A Staged Approach for Contouring of Temporal Deformities Using Porous Polyethylene and Lipofilling. J Craniofac Surg. 2021;32(8):e804-e8. |
|  | Cao J, Zhuang J, Wang C, Jiang D, Su X, Wei Q, et al. Factors Influencing Bone Resorption after Augmentation Mentoplasty with Implants: A Retrospective Study Using Three-Dimensional Imaging. Aesthetic Plast Surg. 2024;48(21):4280-90. |
|  | Machado VF, Chagas RSD, Dos Reis PM, Grillo R. Extrusion of High-density Porous Polyethylene Implants in the Nose. J Craniofac Surg. 2024. |
|  | Konofaos P, Thompson RH, Wallace RD. Long-Term Outcomes With Porous Polyethylene Implant Reconstruction of Large Craniofacial Defects. Ann Plast Surg. 2017;79(5):467-72. |
|  | da Rocha JHF, Ribeiro FCP, Da Silva GD, de Alcantara Giraud RF, Lima RS, Platania F, et al. Short-Term Outcomes of High-Density Porous Polyethylene Orthoses for Chin and Gonial Angle Augmentation. Aesthetic Plast Surg. 2024;48(23):4805-13. |
|  | Wang Y, Zhang Y, Zhang Z, Li X, Pan J, Li J. Reconstruction of Mandibular Contour Using Individualized High-Density Porous Polyethylene (Medpor(®)) Implants Under the Guidance of Virtual Surgical Planning and 3D-Printed Surgical Templates. Aesthetic Plast Surg. 2018;42(1):118-25. |
|  | Song X, Li L, Sun Y, Fan X, Li Z. Long-term infectious complications of using porous polyethylene mesh for orbital fracture reconstruction. Medicine (Baltimore). 2016;95(25):e3819. |
|  | Ridwan-Pramana A, Wolff J, Raziei A, Ashton-James CE, Forouzanfar T. Porous polyethylene implants in facial reconstruction: Outcome and complications. J Craniomaxillofac Surg. 2015;43(8):1330-4. |
|  | Chen CT, Hu TL, Lai JB, Chen YC, Chen YR. Reconstruction of traumatic nasal deformity in Orientals. J Plast Reconstr Aesthet Surg. 2010;63(2):257-64. |
|  | Xu JJ, Teng L, Jin XL, Ji Y, Lu JJ, Zhang B. Porous polyethylene implants in orbital blow-out fractures and enophthalmos reconstruction. J Craniofac Surg. 2009;20(3):918-20. |
|  | Yilmaz M, Vayvada H, Aydin E, Menderes A, Atabey A. Repair of fractures of the orbital floor with porous polyethylene implants. Br J Oral Maxillofac Surg. 2007;45(8):640-4. |
|  | Ozturk S, Sengezer M, Isik S, Turegun M, Deveci M, Cil Y. Long-term outcomes of ultra-thin porous polyethylene implants used for reconstruction of orbital floor defects. J Craniofac Surg. 2005;16(6):973-7. |
|  | Verma S, Garg A, Nastri A. Orbitomaxillary mass after repair of the orbital floor. Br J Oral Maxillofac Surg. 2014;52(10):977-9. |
|  | Prowse SJ, Hold PM, Gilmour RF, Pratap U, Mah E, Kimble FW. Orbital floor reconstruction: a case for silicone. A 12 year experience. J Plast Reconstr Aesthet Surg. 2010;63(7):1105-9. |
|  | Miyasaka M, Tanaka R, Hanai U, Yamazaki A, Iida M, Akamatsu T. A case of chronic infection 28 years after silicone orbital implant. The Tokai journal of experimental and clinical medicine. 2008;33:35-8. |
|  | Huelse R, Freuschle A, Hörmann K, Stuck BA. [Orbitocutaneous fistula after orbital reconstruction]. Hno. 2013;61(4):344-6. |
|  | Morotomi T, Matsunaga K, Kusuhara H, Itani Y, Nakao H, Asamura S, et al. Long-term result of a biodegradable osteo-inductive copolymer for the treatment of orbital blowout fracture. J Craniomaxillofac Surg. 2014;42(5):443-7. |
|  | Eppley BL, Hollier L, Stal S. Hydroxyapatite Cranioplasty: 2. Clinical Experience With a New Quick-Setting Material. Journal of Craniofacial Surgery. 2003;14(2). |
|  | Mathur KK, Tatum SA, Kellman RM. Carbonated apatite and hydroxyapatite in craniofacial reconstruction. Arch Facial Plast Surg. 2003;5(5):379-83. |
|  | Verret DJ, Ducic Y, Oxford L, Smith J. Hydroxyapatite cement in craniofacial reconstruction. Otolaryngol Head Neck Surg. 2005;133(6):897-9. |
|  | Vories A, Mansfield E. Hydroxyapatite cranioplasty in fibrous dysplasia of the skull. Ear Nose Throat J. 2001;80(1):29-31. |
|  | Kinsman M, Aljuboori Z, Ball T, Nauta H, Boakye M. Rapid high-fidelity contour shaping of titanium mesh implants for cranioplasty defects using patient-specific molds created with low-cost 3D printing: A case series. Surg Neurol Int. 2020;11:288. |
|  | Rotaru H, Schumacher R, Kim S-G, Dinu C. Selective laser melted titanium implants: a new technique for the reconstruction of extensive zygomatic complex defects. Maxillofacial Plastic and Reconstructive Surgery. 2015;37(1):1. |
|  | Qassemyar Q, Assouly N, Madar Y, Temam S, Kolb F. Total nasal reconstruction with 3D custom made porous titanium prosthesis and free thoracodorsal artery perforator flap: A case report. Microsurgery. 2018;38(5):567-71. |

Table 7. Excluded Studies and Reasons

| **№** | **Study** | **Reason** |
| --- | --- | --- |
|  | Leão RS, Maior JRS, Lemos CAA, Vasconcelos B, Montes M, Pellizzer EP, et al. Complications with PMMA compared with other materials used in cranioplasty: a systematic review and meta-analysis. Braz Oral Res. 2018;32:e31. | Systematic review and/or meta-analysis |
|  | Oliver JD, Eells AC, Saba ES, Boczar D, Restrepo DJ, Huayllani MT, Sisti A, Hu MS, Gould DJ and Forte AJ: Alloplastic Facial Implants: A Systematic Review and Meta-Analysis on Outcomes and Uses in Aesthetic and Reconstructive Plastic Surgery. Aesthetic Plast Surg 43: 625-636, 2019. doi:10.1007/s00266-019-01370-0 | Systematic review and/or meta-analysis |
|  | Perozzo FAG, Ku YC, Kshettry VR, Sikder P, Papay FA, Rampazzo A, Bassiri Gharb B. High-Density Porous Polyethylene Implant Cranioplasty: A Systematic Review of Outcomes. J Craniofac Surg. 2024 Jun 1;35(4):1074-1079. doi: 10.1097/SCS.0000000000010135. Epub 2024 Apr 29. PMID: 38682928. | Systematic review |
|  | Keyhan SO, Ramezanzade S, Yazdi RG, Valipour MA, Fallahi HR, Shakiba M, Aeinehvand M. Prevalence of complications associated with polymer-based alloplastic materials in nasal dorsal augmentation: a systematic review and meta-analysis. Maxillofac Plast Reconstr Surg. 2022 Apr 22;44(1):17. doi: 10.1186/s40902-022-00344-8. PMID: 35451637; PMCID: PMC9033909. | Systematic review and/or meta-analysis |
|  | AlOtaibi N, Naudi K, Conway D, Ayoub A. The current state of PEEK implant osseointegration and future perspectives: a systematic review. Eur Cell Mater. 2020 Jul 15;40:1-20. doi: 10.22203/eCM.v040a01. PMID: 32667046. | Systematic review |
|  | Khalid SI, Thomson KB, Maasarani S, Wiegmann AL, Smith J, Adogwa O, Mehta AI, Dorafshar AH. Materials Used in Cranial Reconstruction: A Systematic Review and Meta-Analysis. World Neurosurg. 2022 Aug;164:e945-e963. doi: 10.1016/j.wneu.2022.05.073. Epub 2022 May 25. PMID: 35623608. | Systematic review and/or meta-analysis |
|  | Basgul C, Spece H, Sharma N, Thieringer FM, Kurtz SM. Structure, properties, and bioactivity of 3D printed PAEKs for implant applications: A systematic review. J Biomed Mater Res B Appl Biomater. 2021 Nov;109(11):1924-1941. doi: 10.1002/jbm.b.34845. Epub 2021 Apr 15. PMID: 33856114. | Systematic review |
|  | Benato A, Trevisi G, Palombi D, Zeoli F, Sturiale CL. Impact of cement cranioplasty on cerebrospinal fluid leaks after retrosigmoid craniotomy - A systematic review and meta-analysis. J Clin Neurosci. 2025 May;135:111109. doi: 10.1016/j.jocn.2025.111109. Epub 2025 Feb 22. PMID: 39987761.  Rehman U, Shemie M, Sarwar MS, Adebayo O, Brennan PA. Use of biomaterials in the reconstruction of posterior lamellar eyelid defects: a systematic review and meta-analysis. Br J Oral Maxillofac Surg. 2023 Sep;61(7):464-474. doi: 10.1016/j.bjoms.2023.06.001. Epub 2023 Jun 15. PMID: 37400344. | Systematic review and/or meta-analysis |
|  | Di Cosmo L, Pellicanò F, Choueiri JE, Schifino E, Stefini R, Cannizzaro D. Meta-analyses of the surgical outcomes using personalized 3D-printed titanium and PEEK vs. standard implants in cranial reconstruction in patients undergoing craniectomy. Neurosurg Rev. 2025 Mar 21;48(1):312. doi: 10.1007/s10143-025-03470-9. PMID: 40116998. | Meta-analysis |
|  | Henry J, Amoo M, Taylor J, O'Brien DP. Complications of Cranioplasty in Relation to Material: Systematic Review, Network Meta-Analysis and Meta-Regression. Neurosurgery. 2021 Aug 16;89(3):383-394. doi: 10.1093/neuros/nyab180. PMID: 34100535. | Systematic review and/or meta-analysis |
|  | Zhu S, Chen Y, Lin F, Chen Z, Jiang X, Zhang J, Wang J. Complications following titanium cranioplasty compared with nontitanium implants cranioplasty: A systematic review and meta-analysis. J Clin Neurosci. 2021 Feb;84:66-74. doi: 10.1016/j.jocn.2020.12.009. Epub 2020 Dec 28. PMID: 33485602. | Systematic review and/or meta-analysis |
|  | Liu L, Lu ST, Liu AH, Hou WB, Cao WR, Zhou C, Yin YX, Yuan KS, Liu HJ, Zhang MG, Zhang HJ. Comparison of complications in cranioplasty with various materials: a systematic review and meta-analysis. Br J Neurosurg. 2020 Aug;34(4):388-396. doi: 10.1080/02688697.2020.1742291. Epub 2020 Apr 1. PMID: 32233810. | Systematic review and/or meta-analysis |
|  | Abu-Ghname A, Banuelos J, Oliver JD, Vyas K, Daniels D, Sharaf B. Outcomes and Complications of Pediatric Cranioplasty: A Systematic Review. Plast Reconstr Surg. 2019 Sep;144(3):433e-443e. doi: 10.1097/PRS.0000000000005933. PMID: 31461029. | Systematic review |
|  | Oliver JD, Banuelos J, Abu-Ghname A, Vyas KS, Sharaf B. Alloplastic Cranioplasty Reconstruction: A Systematic Review Comparing Outcomes With Titanium Mesh, Polymethyl Methacrylate, Polyether Ether Ketone, and Norian Implants in 3591 Adult Patients. Ann Plast Surg. 2019 May;82(5S Suppl 4):S289-S294. doi: 10.1097/SAP.0000000000001801. PMID: 30973834. | Systematic review |
|  | Punchak M, Chung LK, Lagman C, Bui TT, Lazareff J, Rezzadeh K, Jarrahy R, Yang I. Outcomes following polyetheretherketone (PEEK) cranioplasty: Systematic review and meta-analysis. J Clin Neurosci. 2017 Jul;41:30-35. doi: 10.1016/j.jocn.2017.03.028. Epub 2017 Apr 1. PMID: 28377284. | Systematic review and/or meta-analysis |
|  | Gafar Ahmed M, AlHammad ZA, Al-Jandan B, Almohammadi T, Khursheed Alam M, Bagde H. Silicone Facial Implants, to Fixate or Not to Fixate: A Narrative Review. Cureus. 2023;15(2):e34524. | Review |
|  | Shah AM, Jung H, Skirboll S. Materials used in cranioplasty: a history and analysis. Neurosurgical focus. 2014;36(4):E19. | Review |
|  | Moharil S, Reche A, Durge K. Polyetheretherketone (PEEK) as a Biomaterial: An Overview. Cureus. 2023;15(8):e44307. | Review |
|  | Rojas YA, Sinnott C, Colasante C, Samas J, Reish RG. Facial Implants: Controversies and Criticism. A Comprehensive Review of the Current Literature. Plast Reconstr Surg. 2018;142(4):991-9. | Review |
|  | Jessop ZM, Hague A, Dobbs TD, Stewart KJ, Whitaker IS. Facial Cartilaginous Reconstruction-A Historical Perspective, State-of-the-Art, and Future Directions. Front Surg. 2021;8:680186. | Review |
|  | Quatela VC, Chow J. Synthetic facial implants. Facial Plast Surg Clin North Am. 2008;16(1):1-10, v. | Review |
|  | Kauke-Navarro M, Knoedler L, Knoedler S, Deniz C, Stucki L, Safi AF. Balancing beauty and science: a review of facial implant materials in craniofacial surgery. Front Surg. 2024;11:1348140. | Review |
|  | Han X, Sharma N, Xu Z, Scheideler L, Geis-Gerstorfer J, Rupp F, et al. An In Vitro Study of Osteoblast Response on Fused-Filament Fabrication 3D Printed PEEK for Dental and Cranio-Maxillofacial Implants. J Clin Med. 2019;8(6). | In vitro |
|  | Sarfraz S, Mäntynen PH, Laurila M, Rossi S, Leikola J, Kaakinen M, et al. Comparison of Titanium and PEEK Medical Plastic Implant Materials for Their Bacterial Biofilm Formation Properties. Polymers (Basel). 2022;14(18). | In vitro |
|  | Yu D, Lei X, Zhu H. Modification of polyetheretherketone (PEEK) physical features to improve osteointegration. J Zhejiang Univ Sci B. 2022;23(3):189-203 | In vitro |
|  | Ma R, Tang T. Current strategies to improve the bioactivity of PEEK. Int J Mol Sci. 2014;15(4):5426-45. | In vitro |
|  | Cevik P, Yildirim AZ, Demir Sevinc EH, Gonder A, Kiat-Amnuay S. Using Peek as a Framework Material for Maxillofacial Silicone Prosthesis: An In Vitro Study. Polymers (Basel). 2023 Jun 15;15(12):2694. doi: 10.3390/polym15122694. PMID: 37376341; PMCID: PMC10300953. | In vitro |
|  | Emery BE, Dixit R, Formby CC, Biedlingmaier JF. The resistance of maxillofacial reconstruction plates to biofilm formation in vitro. Laryngoscope. 2003 Nov;113(11):1977-82. doi: 10.1097/00005537-200311000-00023. PMID: 14603059. | In vitro |
|  | Xin H, Ferguson BM, Wan B, Al Maruf DSA, Lewin WT, Cheng K, Kruse HV, Leinkram D, Parthasarathi K, Wise IK, Froggatt C,  Crook JM, McKenzie DR, Li Q, Clark JR. A Preclinical Trial Protocol Using an Ovine Model to Assess Scaffold Implant Biomaterials for Repair of Critical-Sized Mandibular Defects. ACS Biomater Sci Eng. 2024 May 13;10(5):2863-2879. | In vitro |
|  | Kerwell S, Alfaro M, Pourzal R, Lundberg HJ, Liao Y, Sukotjo C, Mercuri LG, Mathew MT. Examination of failed retrieved temporomandibular joint (TMJ) implants. Acta Biomater. 2016 Mar 1;32:324-335. doi: 10.1016/j.actbio.2016.01.001. Epub 2016 Jan 5. PMID: 26768232. | In vitro |
|  | Frake PC, Howell RJ, Joshi AS. Strength of titanium intramedullary implant versus miniplate fixation of mandibular condyle fractures. Otolaryngol Head Neck Surg. 2012 Jul;147(1):33-9. doi: 10.1177/0194599812439391. Epub 2012 Mar 1. PMID: 22394553. | In vitro |
|  | Pietrzak WS, Eppley BL. Antibiotic elution from hydroxyapatite cement cranioplasty materials. J Craniofac Surg. 2005 Mar;16(2):228-33. doi: 10.1097/00001665-200503000-00008. PMID: 15750419. | In vitro |
|  | Zwetyenga N, Catros S, Emparanza A, Deminiere C, Siberchicot F, Fricain JC. Mandibular reconstruction using induced membranes with autologous cancellous bone graft and HA-betaTCP: animal model study and preliminary results in patients. Int J Oral Maxillofac Surg. 2009 Dec;38(12):1289-97. doi: 10.1016/j.ijom.2009.07.018. Epub 2009 Aug 21. PMID: 19699612. | Animal study |
|  | Hobert M, Sharma N, Benzimra C, Hinden S, Oevermann A, Maintz M, Beyer M, Thieringer F, Guevar J. Case report: One-stage craniectomy and cranioplasty digital workflow for three-dimensional printed polyetheretherketone implant for an extensive skull multilobular osteochondosarcoma in a dog. Front Vet Sci. 2024 Aug 29;11:1459272. doi: 10.3389/fvets.2024.1459272. PMID: 39268523; PMCID: PMC11392014. | Animal study |
|  | Ku JK, Kim YK, Yun PY. Influence of biodegradable polymer membrane on new bone formation and biodegradation of biphasic bone substitutes: an animal mandibular defect model study. Maxillofac Plast Reconstr Surg. 2020 Oct 15;42(1):34. doi: 10.1186/s40902-020-00280-5. PMID: 33083383; PMCID: PMC7561620. | Animal study |
|  | Fricia M, Passanisi M, Salamanna F, Parrilli A, Giavaresi G, Fini M. Osteointegration in Custom-made Porous Hydroxyapatite Cranial Implants: From Reconstructive Surgery to Regenerative Medicine. World Neurosurg. 2015 Aug;84(2):591.e11-6. doi: 10.1016/j.wneu.2015.03.027. Epub 2015 Mar 25. PMID: 25819529. | Animal study |
|  | Psarou E, Vezoli J, Schölvinck ML, Ferracci PA, Zhang Y, Grothe I, Roese R, Fries P. Modular, cement-free, customized headpost and connector-chamber implants for macaques. J Neurosci Methods. 2023 Jun 1;393:109899. doi: 10.1016/j.jneumeth.2023.109899. Epub 2023 May 23. PMID: 37230259. | Animal study |
|  | De Meurechy N, Aktan MK, Boeckmans B, Huys S, Verwilghen DR, Braem A, Mommaerts MY. Surface wear in a custom manufactured temporomandibular joint prosthesis. J Biomed Mater Res B Appl Biomater. 2022 Jun;110(6):1425-1438. doi: 10.1002/jbm.b.35010. Epub 2022 Jan 28. PMID: 35088936; PMCID: PMC9306732. | Animal study |
|  | Lin J, Holt-Torres P, Vyas R, Aslani A, Tobin E, Liu H. Magnesium-Based Bioresorbable Implants for Craniomaxillofacial and Orthopaedic Operations: In Vivo Studies in Rats. Journal of the American College of Surgeons. 2021. 233. S208. 10.1016/j.jamcollsurg.2021.07.427. | Animal study |
|  | Namgoong H, Kim MD, Ku Y, Rhyu IC, Lee YM, Seol YJ, Gu HJ, Susin C, Wikesjö UM, Koo KT. Bone reconstruction after surgical treatment of experimental peri-implantitis defects at a sandblasted/acid-etched hydroxyapatite-coated implant: an experimental study in the dog. J Clin Periodontol. 2015 Oct;42(10):960-6. doi: 10.1111/jcpe.12457. Epub 2015 Oct 15. PMID: 26362968. | Animal study |
|  | Chanchareonsook N, Tideman H, Lee S, Hollister SJ, Flanagan C, Jansen JA. Mandibular reconstruction with a bioactive-coated cementless Ti6Al4V modular endoprosthesis in Macaca fascicularis. Int J Oral Maxillofac Surg. 2014 Jun;43(6):758-68. doi: 10.1016/j.ijom.2013.09.014. Epub 2014 Feb 4. PMID: 24507820. | Animal study |
|  | Goh BT, Lee S, Tideman H, Stoelinga PJ. Replacement of the condyle and ascending ramus by a modular endoprosthesis in Macaca fascicularis--part 1: a clinical and radiographic study. J Oral Maxillofac Surg. 2009 Jul;67(7):1392-400. doi: 10.1016/j.joms.2008.11.018. PMID: 19531408. | Animal study |
|  | Oliveira RV, de Souza Nunes LS, Filho HN, de Andrade Holgado L, Ribeiro DA, Matsumoto MA. Fibrovascularization and osteogenesis in high-density porous polyethylene implants. J Craniofac Surg. 2009 Jul;20(4):1120-4. doi: 10.1097/SCS.0b013e3181abb4ab. PMID: 19553849. | Animal study |
|  | Fontana F, Rocchietta I, Dellavia C, Nevins M, Simion M. Biocompatibility and manageability of a new fixable bone graft for the treatment of localized bone defects: preliminary study in a dog model. Int J Periodontics Restorative Dent. 2008 Dec;28(6):601-7. PMID: 19146056. | Animal study |
|  | Bi X, Li M, Zhang Y, Yin M, Che W, Bi Z, et al. Polyetheretherketone (PEEK) as a Potential Material for the Repair of Maxillofacial Defect Compared with E-poly(tetrafluoroethylene) (e-PTFE) and Silicone. ACS Biomater Sci Eng. 2023;9(7):4328-40. | Animal study |
|  | Gosau M, Schiel S, Draenert GF, Ihrler S, Mast G, Ehrenfeld M. [Craniofacial augmentation with porous polyethylene implants (Medpor: first clinical results]. Mund Kiefer Gesichtschir. 2006;10(3):178-84 | Other language |
|  | Poli PP, de Miranda FV, Polo TOB, Santiago Júnior JF, Lima Neto TJ, Rios BR, et al. Titanium Allergy Caused by Dental Implants: A Systematic Literature Review and Case Report. Materials (Basel). 2021;14(18). | Not specific/indirectly relevant |
|  | Thayaparan GK, Lewis PM, Thompson RG, D'Urso PS. Patient-specific implants for craniomaxillofacial surgery: A manufacturer's experience. Ann Med Surg (Lond). 2021;66:102420. | Not specific/indirectly relevant |
|  | Aldabib J. Reinforcement of poly (methyl methacrylate) denture base material. Dental and Medical Journal-Review. 2020;2(2):46-53. | Not specific/indirectly relevant |
|  | Ashraf M, Choudhary N, Kamboh UA, Raza MA, Sultan KA, Ghulam N, et al. Early experience with patient-specific low-cost 3D-printed polymethylmethacrylate cranioplasty implants in a lower-middle-income-country: Technical note and economic analysis. Surg Neurol Int. 2022;13:270. | Not specific/indirectly relevant |
|  | Binhammer A, Jakubowski J, Antonyshyn O, Binhammer P. Comparative Cost-Effectiveness of Cranioplasty Implants. Plast Surg (Oakv). 2020;28(1):29-39. | Not specific/indirectly relevant |
|  | Dondani JR, Iyer J, Tran SD. Surface Treatments of PEEK for Osseointegration to Bone. Biomolecules. 2023;13(3). | Not specific/indirectly relevant |
|  | Rubin JP, Yaremchuk MJ. Complications and toxicities of implantable biomaterials used in facial reconstructive and aesthetic surgery: a comprehensive review of the literature. Plast Reconstr Surg. 1997;100(5):1336-53. | Not specific/indirectly relevant |
|  | Thien A, King NKK, Ang BT, Wang E, Ng I. Comparison of Polyetheretherketone and Titanium Cranioplasty after Decompressive Craniectomy. World Neurosurgery. 2015;83(2):176-80. | Not specific/indirectly relevant |
|  | Siddiqui HA, Pickering KL, Mucalo MR. A Review on the Use of Hydroxyapatite-Carbonaceous Structure Composites in Bone Replacement Materials for Strengthening Purposes. Materials (Basel). 2018;11(10). | Not specific/indirectly relevant |
|  | Podolsky DJ, Mainprize JG, Edwards GP, Antonyshyn OM. Patient-Specific Orbital Implants: Development and Implementation of Technology for More Accurate Orbital Reconstruction. J Craniofac Surg. 2016;27(1):131-3. | Not specific/indirectly relevant |
|  | Mrad MA, Murrad K, Antonyshyn O. Analyzing the Cost of Autogenous Cranioplasty Versus Custom-Made Patient-Specific Alloplastic Cranioplasty. J Craniofac Surg. 2017;28(5):1260-3. | Not specific/indirectly relevant |
|  | Lommen J, Schorn L, Sproll C, Haussmann J, Kübler NR, Budach W, et al. Reduction of CT Artifacts Using Polyetheretherketone (PEEK), Polyetherketoneketone (PEKK), Polyphenylsulfone (PPSU), and Polyethylene (PE) Reconstruction Plates in Oral Oncology. J Oral Maxillofac Surg. 2022;80(7):1272-83. | Not specific/indirectly relevant |
|  | Ku PKM, Vlantis AC, Tong MC, Chan TTT, Yeung ZWC, Cho RHW, et al. A Hybrid Auricular Framework of Autologous Rib Cartilage and a Porous Polyethylene Implant for Reconstruction of Congenital Microtia: A Modification of Nagata's Technique. Facial Plast Surg Aesthet Med. 2024;26(1):15-22. | Not specific/indirectly relevant |

Table 8. Risk of bias assessment of case reports (JBI critical appraisal checklist for case reports)

| Authors name, publication year | Were patient’s demographic characteristics clearly described? | Was the patient’s history clearly described and presented as a timeline? | Was the current clinical condition of the patient on presentation clearly described? | Were diagnostic tests or assessment methods and the results clearly described? | Was the intervention(s) or treatment procedure(s) clearly described? | Was the post-intervention clinical condition clearly described? | Were adverse events (harms) or unanticipated events identified and described? | Does the case report provide takeaway lessons? | Overall | Used material |
| --- | --- | --- | --- | --- | --- | --- | --- | --- | --- | --- |
| Narciso R. et al. (2021) | Yes | Yes | Yes | Yes | Yes | Yes | No | Yes | Include | PEEK |
| Nocini R. et al. (2022) | Yes | Yes | Yes | Yes | Yes | Yes | No | Yes | Include | PEEK |
| Shi H. et al. (2022) | Yes | Yes | Yes | Yes | Yes | Yes | No | Yes | Include | PEEK |
| Hamsho R. et al. (2022) | Yes | Yes | Yes | Yes | Yes | Yes | No | Yes | Include | PEEK |
| Long J. et al. (2023) | Yes | Yes | Yes | Yes | Yes | Yes | No | Yes | Include | PEEK |
| Khashaba M. et al. (2023) | Yes | Yes | Yes | Yes | Yes | Yes | Yes | Yes | Include | PEEK |
| Liu B. et al. (2019) | Yes | Yes | Yes | Yes | Yes | Yes | Yes | Yes | Include | PEEK |
| van der Wel H. et al. (2024) | Yes | Yes | Yes | Yes | Yes | Yes | No | Yes | Include | PEEK |
| Pöppe JP. et al. (2024) | Yes | Yes | Yes | Yes | Yes | Yes | No | Yes | Include | PEEK |
| Komal S. et al. (2025) | Yes | Yes | Yes | Yes | Yes | Yes | Yes | Yes | Include | PEEK |
| Habib LA. et al. (2021) | Yes | Yes | Yes | Yes | Yes | Yes | Yes | Yes | Include | PEEK, Polyethelen |
| Dib J. et al. (2018) | Yes | Yes | Yes | Yes | Yes | Yes | Yes | Yes | Include | PMMA |
| Milhomem AC. et al. (2018) | Yes | Yes | Yes | Yes | Yes | Yes | Yes | Yes | Include | PMMA |
| Puricelli E. et al. (2022) | Yes | Yes | Yes | Yes | Yes | Yes | Yes | Yes | Include | PMMA |
| da Silva de Menezes JD. et al. (2017) | Yes | Yes | Yes | Yes | Yes | Yes | Yes | Yes | Include | Polyethelen |
| Carloni R. et al.(2016) | Yes | Yes | Yes | Yes | Yes | Yes | Yes | Yes | Include | HA |
| Vories A. et al. (2001) | Yes | Yes | Yes | Yes | Yes | Yes | No | Yes | Include | HA |
| Morice A. et al. (2017) | Yes | Yes | Yes | Yes | Yes | Yes | Yes | Yes | Include | HA |
| Chattopadhyay C. et al. (2019) | Yes | Yes | Yes | Yes | Yes | Yes | Yes | Yes | Include | Titanium |
| Touré G. et al. (2019) | Yes | Yes | Yes | Yes | Yes | Yes | No | Yes | Include | Titanium |
| Melville JC. et al. (2019) | Yes | Yes | Yes | Yes | Yes | Yes | No | Yes | Include | Titanium |
| Woo JM. et al. (2018) | Yes | Yes | Yes | Yes | Yes | Yes | No | Yes | Include | Titanium |
| Hatamleh MM. et al. (2016) | Yes | Yes | Yes | Yes | Yes | Yes | No | Yes | Include | Titanium |
| Sesqué A. et al. (2021) | Yes | Yes | Yes | Yes | Yes | Yes | No | Yes | Include | Titanium |
| Leiser Y. et al. (2016) | Yes | Yes | Yes | Yes | Yes | Yes | No | Yes | Include | Titanium |
| Lee U-L. et al. (2016) | Yes | Yes | Yes | Yes | Yes | Yes | No | Yes | Include | Titanium |
| Yates JM. et al. (2009) | Yes | Yes | Yes | Yes | Yes | Yes | No | Yes | Include | Titanium |
| Al-Sukhun J. et al. (2023) | Yes | Yes | Yes | Yes | Yes | Yes | No | Yes | Include | Titanium |
| Watson J. et al. (2014) | Yes | Yes | Yes | Yes | Yes | Yes | No | Yes | Include | Titanium |
| Vrielinck L. et al. (2014) | Yes | Yes | Yes | Yes | Yes | Yes | No | Yes | Include | Titanium |
| Han X. et al. (2019) | Yes | Yes | Yes | Yes | Yes | Yes | No | Yes | Include | Titanium |
| Kundakçioğlu A. et al. (2024) | Yes | Yes | Yes | Yes | Yes | Yes | No | Yes | Include | Titanium |
| Rotaru H. et al. (2015) | Yes | Yes | Yes | Yes | Yes | Yes | Yes | Yes | Include | Titanium |
| Li P. et al. (2014) | Yes | Yes | Yes | Yes | Yes | Yes | No | Yes | Include | Titanium |
| Qassemyar Q. et al. (2018) | Yes | Yes | Yes | Yes | Yes | Yes | No | Yes | Include | Titanium |
| Miyasaka M. et al. (2008) | Yes | Yes | Yes | Yes | Yes | Yes | No | Yes | Include | Silicone |
| Verma S. et al. (2014) | Yes | Yes | Yes | Yes | Yes | Yes | No | Yes | Include | Silicone |
| Huelse R. et al. (2013) | Yes | Yes | Yes | Yes | Yes | Yes | No | Yes | Include | Silicone |

Table 9. Risk of bias assessment of case series (JBI critical appraisal checklist for case series)

| Authors name, publication year | Were there clear criteria for inclusion in the case series? | Was the condition measured in a standard, reliable way for all participants included in the case series? | Were valid methods used for identification of the condition for all participants included in the case series? | Did the case series have consecutive inclusion of participants? | Was there clear reporting of the demographics of the participants in the study? | Was there clear reporting of clinical information of the participants? | Were the outcomes or follow up results of cases clearly reported? | Was there clear reporting of the presenting site(s)/clinic(s) demographic information? | Was statistical analysis appropriate? | Overall appraisal | Used material |
| --- | --- | --- | --- | --- | --- | --- | --- | --- | --- | --- | --- |
| Kim MM. et al. (2009) | Yes | Yes | Yes | No | Yes | Yes | Yes | No | Not applicable | include | PEEK |
| Gerbino G. et al. (2013) | Yes | Yes | Yes | Yes | Yes | Yes | Yes | Yes | Yes | include | PEEK |
| Jalbert F. et al. (2014) | Yes | Yes | Yes | Yes | Yes | Yes | Yes | Yes | Yes | include | PEEK |
| Eolchiyan SA. (2014) | Yes | Yes | Yes | Yes | Yes | Yes | Yes | Yes | Yes | include | PEEK, titanium |
| Alonso-Rodriguez E. et al. (2015) | Yes | Yes | Yes | Yes | Yes | Yes | Yes | Yes | Yes | include | PEEK |
| Sainsbury DC. et al. (2017) | Yes | Yes | Yes | Yes | Yes | Yes | Yes | Yes | Yes | include | PEEK |
| Alasseri N. et al. (2020) | Yes | Yes | Yes | Yes | Yes | Yes | Yes | Yes | Yes | include | PEEK, titanium |
| Yang M. et al. (2021) | Yes | Yes | Yes | No | Yes | Yes | Yes | No | Not applicable | include | PEEK |
| Li Y. et al. (2022) | Yes | Yes | Yes | Yes | Yes | Yes | Yes | No | Yes | include | PEEK |
| Cárdenas-Serres C. et al. (2024) | Yes | Yes | Yes | Yes | Yes | Yes | Yes | Yes | Yes | include | PEEK |
| Dean A. et al. (2024) | Yes | Yes | Yes | No | Yes | Yes | Yes | No | Not applicable | include | PEEK |
| Groth MJ. et al. (2006) | Yes | Yes | Yes | Yes | Yes | Yes | Yes | No | Yes | include | PMMA |
| Bassi M. et al. (2021) | Yes | Yes | Yes | Yes | Yes | Yes | Yes | No | Yes | include | PMMA |
| Englar KM. et al. (2022) | Yes | Yes | Yes | Yes | Yes | Yes | Yes | Yes | Yes | include | PMMA |
| Martinez-Seijas P. et al. (2018) | Yes | Yes | Yes | Yes | Yes | Yes | Yes | No | Yes | include | PMMA |
| Landry M. et al. (2021) | Yes | Yes | Yes | No | Yes | Yes | Yes | No | No | include | Polyethelen |
| Kattimani VS. et al. (2016) | Yes | Yes | Yes | Yes | Yes | Yes | Yes | Yes | No | include | HA |
| Systermans S. et al. (2024) | Yes | Yes | Yes | Yes | Yes | Yes | Yes | No | Yes | include | HA |
| Verbist M. et al. (2024) | Yes | Yes | Yes | No | Yes | Yes | Yes | Yes | No | include | HA |
| Dediol E. et al. (2013) | Yes | Yes | Yes | Yes | Yes | Yes | Yes | Yes | No | include | Titanium |
| Chen ST. et al. (2015) | Yes | Yes | Yes | Yes | Yes | Yes | Yes | Yes | Yes | include | Titanium |
| Cortese A. et al. (2023) | Yes | Yes | Yes | No | Yes | Yes | Yes | Yes | Yes | include | Titanium |
| Mounir M.et al. (2020) | Yes | Yes | Yes | Partial | Yes | Yes | Yes | Partial | Yes | include | Titanium |
| Helmers R. et al. (2021) | Yes | Yes | Yes | Yes | Yes | Yes | Yes | Yes | Yes | include | Titanium |
| Wei LA. et al. (2016) | Yes | Yes | Yes | Yes | Yes | Yes | Yes | Yes | Yes | include | Titanium |
| Daniel E. et al. (2004) | Yes | Yes | Yes | Yes | Yes | Yes | Yes | Yes | Yes | include | Titanium |
| Xue R. et al. (2019) | Yes | Yes | Yes | Yes | Yes | Yes | Yes | Yes | Yes | include | Titanium |
| Henry A. et al. (2020) | Yes | Yes | Yes | Yes | Yes | Yes | Yes | Yes | Yes | include | Titanium |
| Kinsman M. et al. (2020) | Yes | Yes | Yes | Yes | Yes | Yes | Yes | Yes | Yes | include | Titanium |
| Yim HW. et al. (2015) | Yes | Yes | Yes | Yes | Yes | Yes | Yes | Yes | Yes | include | Silicone |

Table 10. Risk of bias assessment of non-randomized studies (ROBINS-I tool)


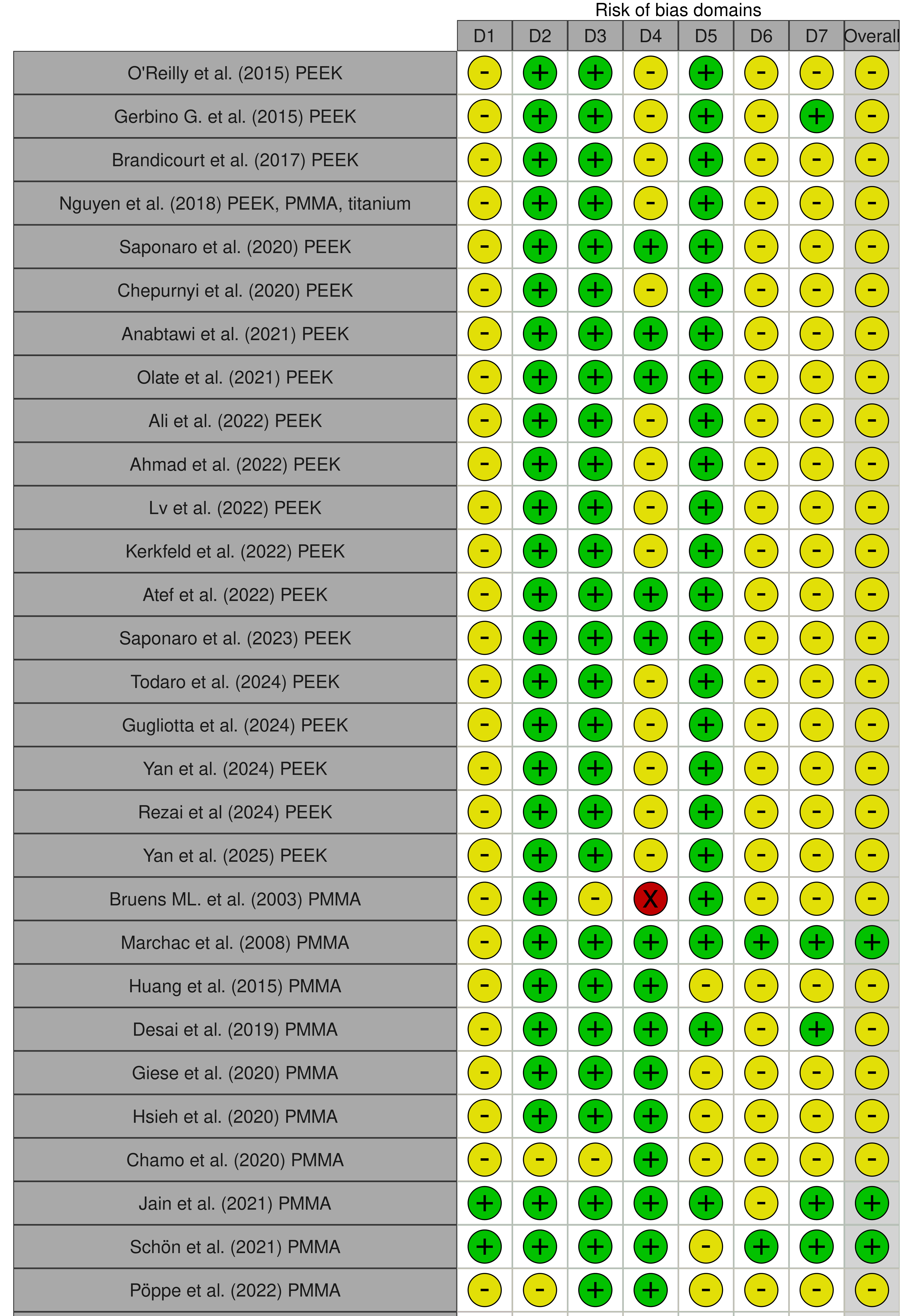


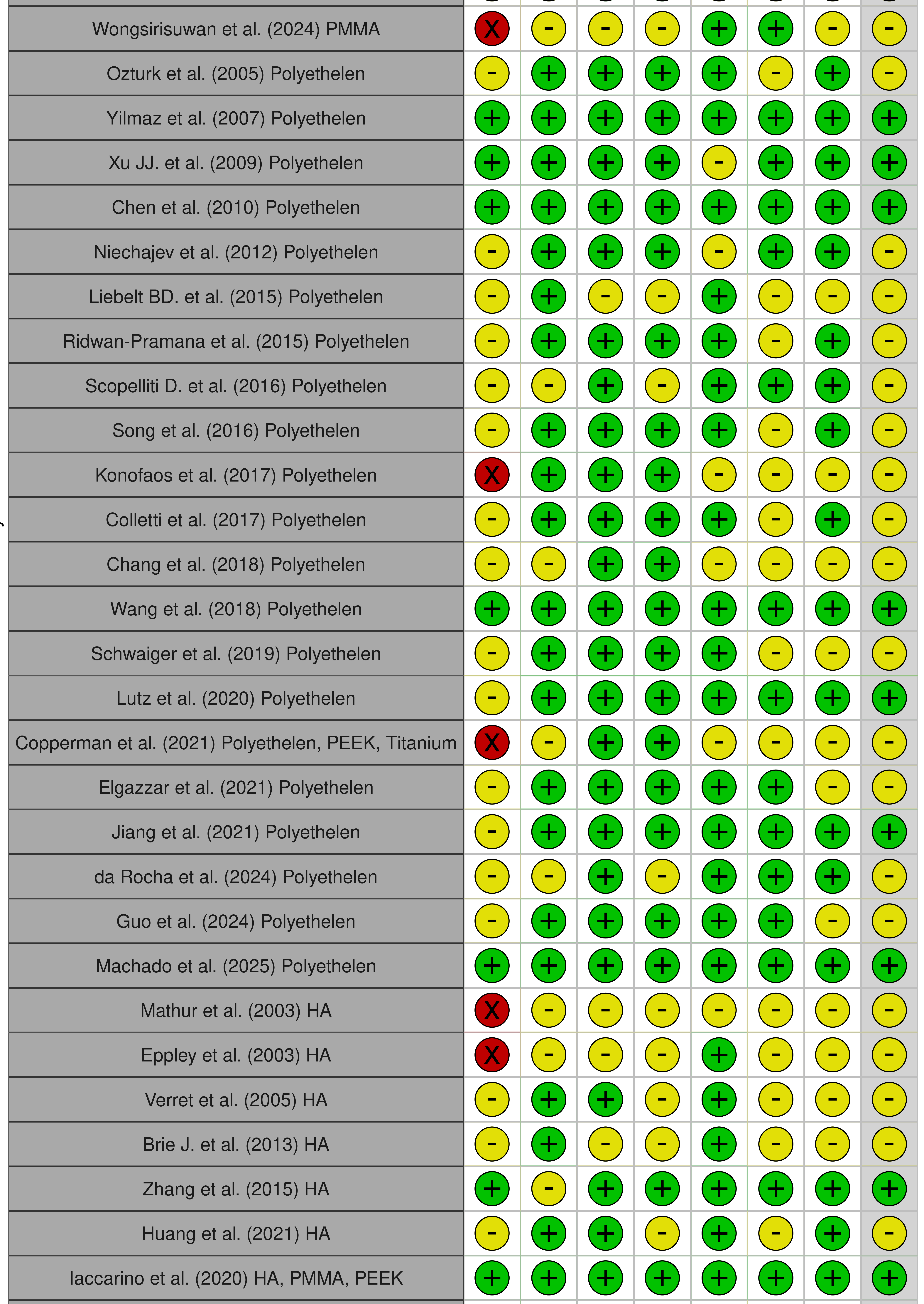


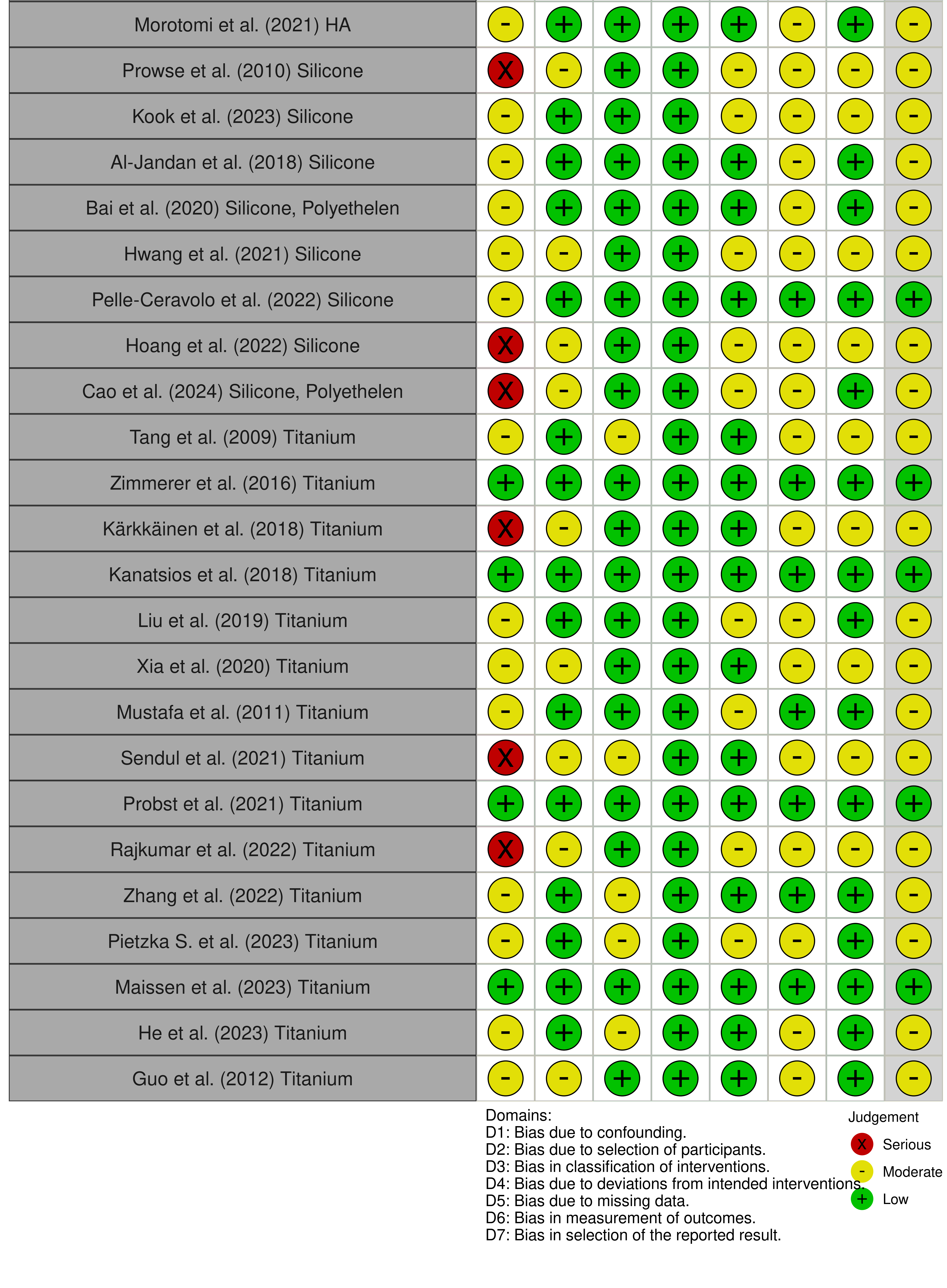

Supplement: Supplementary file 1 — Supplementary Material 1. [file 40902_2025_482_MOESM1_ESM.docx]
