# Supplementary material for: Polyetheretherketone for craniomaxillofacial defects: cases report, evaluation of patients’ satisfaction and a systematic literature review
Source: Maxillofac Plast Reconstr Surg. 2025 Oct 24;47(1):36. doi: 10.1186/s40902-025-00482-9 (PMC12552202; doi:10.1186/s40902-025-00482-9)
Supplement: Supplementary file 2 — Supplementary Material 2. [file 40902_2025_482_MOESM2_ESM.docx]

**Appendix B**

Table A1. Summary of the characteristics of the analyzed studies with complications associated with different implant materials (n = 83)

| **N** | **Author** | **Publication year** | **Study type** | **Population (sample size)** | **Area/region of implant use** | **Follow-up period** | **Clinical outcomes** | **Complications** | **Implant material** |
| --- | --- | --- | --- | --- | --- | --- | --- | --- | --- |
| 1 | Khashaba M. et al. | 2023 | Case report | 1 | Temporal region | 5 years | Improved function and cosmetics, minor skin stretching over implant after 5 years | Minor skin stretching over implant after 5 years | PEEK |
| 2 | Olate S. et al. | 2021 | Retrospective study | 21 | Mandibular angle | 6 months | Good stability and facial symmetry, better surgical efficiency with PEEK PSI | Two cases of infection with PEEK implants, managed with antibiotics | PEEK |
| 3 | Saponaro G. et al. | 2020 | Retrospective study | 15 | Mandibular angle - 8, fronto-orbital region - 7 | 7 months to 4 years | Significant facial symmetry improvement, acceptable esthetic results | One case of imperfect implant fitting requiring adjustment | PEEK |
| 4 | Rezai A. et al. | 2024 | Retrospective study | 11 | Orbit+cranio vault | 6 months | Improved exophthalmos, good aesthetic and oncological outcomes, some complications | 4 cases: hematoma - 1, impaired wound healing - 2, postoperative visual acuity deterioration -1 | PEEK |
| 5 | Todaro M. et al. | 2024 | Retrospective study | 22 | Mandible angle - 5, fronto-orbital 7, vault - 10 | 1-4 years | Bone regeneration observed in most cases, some bone resorption noted | bone resorption - 1 | PEEK |
| 6 | Ali S. et al. | 2022 | Prospective study | 8 | Temporal | 18 months | Good esthetic outcome, only one case of seroma | One case of seroma, resolved with aspiration | PEEK |
| 7 | Anabtawi M. et al. | 2021 | Retrospective review | 10 | Zygoma -5, mandible - 5 | 11-61 months | Stable implants, no exposure or displacement | Three cases of complications: edema, nerve paresthesia, sinusitis | PEEK |
| 8 | Kim M.M. et al. | 2009 | Case series | 4 | Orbit+maxilla - 2 cases, orbitocranial - 1, anteriot table of frontal sinus - 1 | 14 to 20 months | All had excellent postoperative aesthetic and functional results | 1 case - dehiscence | PEEK |
| 9 | Ahmad AF. et al. | 2022 | Cohort study | 10 | Chin - 4, mandibular angle - 2, zygoma - 2, orbit - 1, nasomaxillary region - 1 | 3 months | High patient satisfaction except one case requiring removal | One implant removal due to infection | PEEK |
| 10 | Li Y. et al. | 2022 | Case series | 6 | Mandible defects (symphysis to condyle) | 10-24 months | Successful reconstruction with good function, one case of implant exposure | One implant removal due to exposure at 10 months | PEEK |
| 11 | Cárdenas-Serres C. et al | 2024 | Case series | 15 | Cranioorbital | 2.5 years | Successful reconstructions, good functional and aesthetic outcomes | One implant replacement due to positioning error | PEEK |
| 12 | Gugliotta Y. et al. | 2024 | Retrospective study | 37 | Zygoma-2, frontal+zygoma - 2, ZMC - 1, temporal+zygoma - 4, zygoma+mandible angle - 2, frontal - 5, frontotemporal - 3, frontal+orbit - 3, temporal - 9, parietotemporal - 1, temporal+orbit - 7, mandible body - 3, mandibular angle - 2 | 78.6 months | High patient satisfaction, improved aesthetics and function | 16 complications in 13 patients (seroma - 9, dehiscence - 4, infection - 3) | PEEK |
| 13 | Kerkfeld V. et al. | 2022 | Retrospective study | 8 | Mandible, maxilla, temporal, zygoma, periorbital | 9 months | Improved facial symmetry, high patient satisfaction | Intraoral exposure of PEEK implants in 2 patients | PEEK |
| 14 | Alonso-Rodriguez E. et al. | 2015 | Case series | 14 | ZMC - 4, frontal - 4, orbit -2, vault - 3, zygoma+temporal -1 | 28.6 months | Highly satisfactory aesthetic results | 2 infections (one requiring implant removal), 1 seroma, 1 CSF leak | PEEK |
| 15 | Yan S. et al. | 2025 | Retrospective study | 24 | Mandibular angle | 6.6 months | High surgical accuracy, significant aesthetic improvements | 8 cases of temporary sensory disturbance | PEEK |
| 16 | Komal S. et al. | 2025 | Case report | 1 | Fronto-ethmoidal, orbital | 8 months | Successful reconstruction initially, implant removal due to infection | PEEK implant infection requiring removal | PEEK |
| 17 | Jalbert F. et al. | 2014 | Case series | 5 | Fronto-orbital, craniofacial | up to 2 years | Good cosmetic and functional results, improved symmetry | None major; two cases of temporary diplopia | PEEK |
| 18 | O’Reilly EB. et al. | 2015 | Retrospective study | 19 | Vault | 24 to 106 months (average 59 months) | Improved anatomic accuracy, mirror image aesthetics, and time-saving reconstruction | 3 cases required implant removal due to infection-1, exposure-1, poor cosmetic outcome/step-off-1 | PEEK |
| 19 | Brandicourt P. et al. | 2017 | Retrospective study | 37 | Vault | 4.3 years (range 2 months to 9 years) | High patient satisfaction, few complications | 1 infection (2.7%) requiring removal, 6 cases (16%) required fat grafting for temporal projection correction | PEEK |
| 20 | Nguyen PD. et al. | 2018 | Retrospective study | 72 | Vault | 30 months | Effective reconstruction, high success rate | 6 cases of infection in PEEK group 15 cases of dural tear (PEEK= 4, PMMA = 9, titanium = 2) 3 cases of seroma (PEEK=2, PMMA=1) 5 cases of implants removal (1 late exposure of titanium implant: 3 cases of infection in PEEK group; 1 case of late fracture of PMMA implant) 7 cases of headache (PEEK=3, PMMA=3, titanium=1) 1 case of dehiscence (titanium) | PEEK (n=72), PMMA (n=42), and titanium (n=22) implants |
| 21 | Sainsbury DC. et al. | 2017 | Case series | 3 | Zygoma | 12 months | Successful reconstruction, aesthetic improvements | One case of transient anisocoria due to globe compression (corrected in redesign) | PEEK |
| 22 | Copperman TS. et al. | 2021 | Retrospective study | 1 | Orbit | 9–30 months | Improved facial symmetry and function | Two cases of postoperative infection (Polyethylene) | Porous Polyethylene, PEEK, Titanium |
| 23 | Chen ST. et al. | 2015 | Retrospective review | 7 | Vault | 28.57 months | Excellent aesthetic and functional outcomes | Subclinical infections in 2 patients, resolved with antibiotics | Titanium |
| 24 | Mounir M. et al. | 2020 | Case series | 7 | Mandible | 3-5 years | Good occlusion, masticatory function, mouth opening, and facial esthetics in 4 patients | Three implant failures due to mucosal dehiscence and persistent fistulae | Titanium |
| 25 | He Q. et al. | 2023 | Pilot study | 10 | Orbit | 1 month | Complete recovery of SOFS symptoms, restored orbital volume | One patient had persistent infraorbital nerve hypoesthesia for several months | Titanium |
| 26 | Helmers R. et al. | 2021 | Case series | 2 | Frontal sinus | Not specified | Accurate reconstruction with rapid recovery and no complications | Transient hypoesthesia | Titanium |
| 27 | Maissen M. et al. | 2023 | Retrospective study | 58 | Orbit | 5 months | Better ophthalmologic outcomes with primary reconstruction | Diplopia persisted in 13 patients | Titanium |
| 28 | Sendul SY. et al. | 2021 | Retrospective study | 11 | Orbit | 10–48 months | Successful rehabilitation in 9 patients | Implant loss in 4 patients, local infections in 3 | Titanium |
| 29 | Tang W. et al. | 2009 | Retrospective study | 51 | Temporomandibular joint | 2-5 years | Improved jaw function, reduced joint pain, and esthetic improvement | Fewer complications and reoperations with titanium implants | Titanium |
| 30 | Mustafa SF. et al. | 2011 | Retrospective review | 22 | Orbit | up to 2 years | Significant improvement in orbital volume and function | One case required ocular muscle surgery, no major complications | Titanium |
| 31 | Guo J. et al. | 2012 | Retrospective study | 102 | Temporal region | 6-24 months | Successful reconstruction with improved temporal contour | No infections, minimal complications | Titanium |
| 32 | Dediol E. et al. | 2013 | Case series | 5 | Maxilla | 12 months | Successful midface projection, globe position, and facial height restoration | One case of mesh exposure managed successfully | Titanium |
| 33 | Kanatsios S. et al. | 2018 | Retrospective study | 60 | Temporomandibular joint | 2-9 years | Significant pain reduction and improved jaw function | One prosthesis failure over 9 years | Titanium |
| 34 | Liu BY. et al. | 2019 | Retrospective study | 12 | Maxilla | 4-12 months | Successful reconstruction with restored orbital volume and facial symmetry | Two cases of titanium mesh exposure, four cases of limited mouth opening | Titanium |
| 35 | De Meurechy NK. et al. | 2020 | Systematic review & Meta-analysis | 15 | Temporomandibular joint | 1-3 years | Both stock and custom TMJ replacements improve function and pain | Custom TMJ implants reduce morbidity and failure risk | Titanium |
| 36 | Cortese A. et al. | 2023 | Case report | 2 | Mandible | 1 year | Successful reconstruction, functional TMJ preserved | Potential risks with prosthesis contamination | Titanium |
| 37 | Han X. et al. | 2023 | Case report | 1 | Mandibular condyle | 6 years | No tumor recurrence, good occlusion and TMJ function | Slight facial asymmetry | Titanium |
| 38 | Rajkumar A., Sidebottom A.J. | 2022 | Prospective cohort study | 42 | Temporomandibular joint | 10 years | Significant pain relief, improved function, high implant survival rate | Two failures (one due to infection, one due to reankylosis) | Titanium |
| 39 | Kundakçıoğlu A. et al. | 2024 | Case report | 1 | Maxilla | 45 days | Successful reconstruction, improved aesthetics and function | Minimal complications | Titanium |
| 40 | Pöppe JP. et al. | 2022 | Retrospective study | 14 | Vault | 2-4 weeks | Excellent cosmetic results | Three cases of postoperative hematoma | PMMA |
| 41 | Huang GJ. et al. | 2015 | Retrospective study | 20 | Vault | 34.2 ± 22.2 weeks | Low complication rate | Two cases of temporal hollowing | PMMA |
| 42 | Chamo D. et al. | 2020 | Retrospective study | 10 | Vault | Not specified | High accuracy of implant reproduction | Minimal deviations in implant accuracy | PMMA |
| 43 | Ming-Chi Hsieh A. et al. | 2020 | Retrospective study | 15 | Vault | 3-36 months (average of 11.4 months) | High replicability of planned results | One case of wound dehiscence, one mild hematoma | PMMA |
| 44 | Giese H. et al. | 2020 | Retrospective study | 67 | Vault | 39.7 months | Good quality of life and cosmetic outcome | epidural/subdural hemorrhage (3), calcified subdural hematoma (1), healing disorders (3), graft infection (3). | PMMA |
| 45 | Jain R. et al. | 2021 | Retrospective study | 25 | Vault (1), zygoma (15), orbit (7, chin - 4, mandible - 1, forehead - 4, nose - 1, temporal -1 | 12 months | Good to excellent aesthetic outcome | One case of implant exposure | PMMA |
| 46 | Schön SN. et al. | 2021 | Case series | 16 | Vault | 6 months | Well-fitting implants, good cosmetic results | hematoma (3), fluid collection (1), skin flap necrosis (1) | PMMA |
| 47 | Wongsirisuwan M. | 2024 | Prospective study | 19 | Anterior temporal | 6 months | Significant esthetic improvement | One case of wound infection | PMMA |
| 48 | Desai JB. | 2019 | Retrospective study | 30 | Vault | Not specified | High patient satisfaction, better fit with 3D printed implants | 13.3% infection rate - 4 cases | PMMA |
| 49 | Marchac D., Greensmith A. | 2008 | Retrospective study | 98 | Vault | Minimum 3 years, average 7.1-8.2 years | 89/98 patients retained implants, 85 had excellent or good results | 3 cases - infection, 1 case - implant fracture | PMMA |
| 50 | Elgazza K., Elshahat A. | 2021 | Prospective study | 8 | Temporal | 1 to 8 years | Improved aesthetic outcome, high patient satisfaction | 2 patients needed an additional lipofilling session | Polyethylene |
| 51 | Cao J. et al. | 2024 | Retrospective study | 28 | Chin | 5 years | Higher bone resorption in silicone implants compared to Medpor | 13 cases of bone resorbtion | Silicone, Polyethylene |
| 52 | Machado VF. et al. | 2025 | Case series | 23 | Nasal columella, nasal tip, septal region | Not specified | High incidence of implant exposure, infections, and extrusion; all cases required implant removal | 19 cases of implant exposure, chronic inflammation, and postoperative pain | Polyethylene |
| 53 | Lutz JC. et al. | 2020 | Retrospective study | 24 | chin (3), zygoma (8), mandible (13) | 41 months (mean) | Satisfactory aesthetic outcomes, improved midface and chin projection | 2 cases of asymmetry | Polyethylene |
| 54 | Jiang C. et al. | 2021 | Clinical study | 32 | Auricular (Ear) | 6-24 months | Improved hearing and aesthetic outcomes in microtia patients | auricle stent fracture, 2 patients developed local ear infections | Polyethylene |
| 55 | Schwaiger, M. et al. | 2019 | Clinical study | 51 | Paranasal (63), zygoma (38), nasal dorsal (20), orbit (1) | mean follow-up period 34.2 months (range 1 - 106 months) | Effective for midface augmentation in cleft patients | 5 patients: Infection occurred in 3.3% of all inserted implants (4/122), leading to removal of the implant in 2.5% (3/122). | Polyethylene |
| 56 | Guo JS. et al. | 2024 | Retrospective review | 38 | Chin | 2 year | High patient satisfaction, effective chin augmentation | 15.8% temporary nerve numbness (6), 2.6% wound infection (1) | Polyethylene |
| 57 | Landry M. et al. | 2021 | Case report | 2 | Maxilla | 3-6 years | Successful reconstructions, but risk of delayed infections | Delayed infections, implant removal (2) | Polyethylene |
| 58 | Konofaos P. et al. | 2017 | Retrospective study | 18 | Forehead (6), orbit (4), parietal (4), temporal (4) | 2+ years | Low infection rate, high durability | Implant exposure in 3 patients (15%) | Polyethylene |
| 59 | da Rocha JHF. et al. | 2024 | Observational study | 100 | chin (56), mandible angle (17), chin and mandible (39) | 30 days | High patient satisfaction, transient paresthesia | 1 case of suture dehiscence, 3 wound infections, 4 transient cases of paresthesia | Polyethylene |
| 60 | Wang Y. et al. | 2018 | Retrospective study | 12 | Mandible | 7-14 months | Accurate reconstruction, satisfactory aesthetic results | 1 case of infection leading to implant removal | Polyethylene |
| 61 | Song X. et al. | 2016 | Retrospective study | 21 | Orbit | 3-45 months | Majority recovered, some cases of persistent infections | Infections, poor vascularization, abscess formation - 19 cases, edema and hyperemia - 2 cases | Polyethylene |
| 62 | Ridwan-Pramana A. et al. | 2015 | Retrospective study | 40 | Mandible (11), orbit (14), zygoma (13), chin (2) | 4-96 months (mean 25.4 months) | Low complication rate, high patient satisfaction | infection - 5 cases, fistula - 1 case | Polyethylene |
| 63 | Niechajev I. | 2012 | Prospective study | 102 | Nose (61), chin (33), zygoma (6), maxilla (2) | 6 months - 15 years (median 7 years) | High patient satisfaction, stable results over long term | Infections in 3 rhinoplasty cases, partial extrusion in 2 cases | Polyethylene |
| 64 | Chen CT. et al. | 2010 | Retrospective study | 32 | Nose | 25.4 months | 90.6% aesthetic improvement, 84.4% patient satisfaction | 3 complications: 1 implant-tip exposure, 2 infections | Polyethylene |
| 65 | Xu JJ.et al. | 2009 | Retrospective study | 68 | Orbit | 2 months - 8 years | Effective in restoring orbital volume and function | Persistent diplopia (9 cases), one overcorrection requiring revision | Polyethylene |
| 66 | Yilmaz M. et al. | 2007 | Retrospective study | 26 | Orbit | 6-24 months | High correction rate of enophthalmos and diplopia, stable implant position | Postoperative infection (4 cases), persistent ectropion (2 cases) | Polyethylene |
| 67 | Ozturk S. et al. | 2005 | Prospective study | 38 | Orbit | 5 months - 6 years | Durable reconstruction, mimics orbital floor anatomy | Persistent enophthalmos (3 cases), ectropion (3 cases, one required correction) | Polyethylene |
| 68 | Verma S. et al. | 2014 | Case report | 1 | Orbit | 6 months | Improved enophthalmos, resolved diplopia | Inclusion cyst formation after 31 years | Silicone |
| 69 | Prowse SJ. et al. | 2010 | Retrospective study | 58 | Orbit | 63 months (mean) | High patient satisfaction, low complication rates | extrusion - 3 cases, infection - 1 case, diplopia - 1 case | Silicone |
| 70 | Miyasaka M. et al. | 2008 | Case report | 1 | Orbit | 28 years | Resolved orbital pain and diplopia after implant removal | Chronic infection, residual silicone implant found after 28 years | Silicone |
| 71 | Al-Jandan B., Marei HF. | 2018 | Prospective study | 58 | Mandible | 6 months | Low infection and displacement rates, high patient satisfaction | 5 cases of infection, 16 cases of displacement, dissatisfaction - 3 | Silicone |
| 72 | Hoang TA. et al. | 2022 | Retrospective cohort study | 38 | Nose | 6 months | Improved nasal symmetry, higher nasal dorsum, increased patient satisfaction | 7.89% overall complication rate (2 infections, 1 dorsal implant displacement) | Silicone, Polyethylene, auricular cartilage |
| 73 | Park JH. et al. | 2021 | Retrospective study | 79 | Chin | 12.3 months (mean) | High patient satisfaction with aesthetic results | Four revision cases due to infection (2 cases) or wound dehiscence (2 cases) | Silicone |
| 74 | Huelse R. et al. | 2013 | Case report | 1 | Orbit | 15 years | Symptoms resolved after implant removal and reconstruction with titanium plate | Orbitocutaneous fistula, implant displacement, chronic inflammation | Silicone |
| 75 | Kook WS. et al. | 2023 | Prospective study | 56 | Nose | 1 year (minimum) | Improved nasal contour, reduced contracture deformities, high patient satisfaction | 1 case of infection, 3 cases of excessive resorption, 2 cases of displacement | Silicone |
| 76 | Pelle-Ceravolo M. et al. | 2022 | Retrospective study | 350 | Zygoma | Mean 28 months (range 5 months - 28 years) | High patient satisfaction, improved midface rejuvenation | intraoral exrusion -1 case, hematoma - 1 case, seroma - 5 cases | Silicone |
| 77 | Systermans S. et al. | 2024 | Case series | 13 | Mandible (3), zygoma (3), orbit (2), chin (5) | 1-22 months (Mean 9 months) | Osteointegration observed at 6 months and 1 year; no infections or displacements | One case of implant exposure leading to removal | HA |
| 78 | Huang Q. et al. | 2021 | Retrospective review | 15 | Vault | 0.5-4 years (Mean 2.2 years) | Successful skull defect reconstruction; no infections or implant exposure | One case of seroma, managed non-operatively | HA/Epoxide acrylate maleic |
| 79 | Zhang L. et al. | 2015 | Retrospective study | 12 | ZMC (6), zygoma (2), mandible (3), vault (1) | 10-69 months | Successful reconstruction; good symmetry; no facial nerve weakness | One case of infection leading to implant removal | HA/Epoxide acrylate maleic |
| 80 | Eppley BL. et al. | 2003 | Clinical study | 62 | Vault | 2 years | Successful cranioplasty with rapid setting; good osteoconduction | 3 cases of infection (5%) requiring implant removal | HA cement |
| 81 | Mathur KK. et al. | 2003 | Comparative study | 13 | Frontal bone (6), orbit (4), mandible (1), temporal (1), complex (1) | 2-48 months | Successful reconstruction; good tissue compatibility | 6 cases (17%) of infection requiring implant removal | Carbonated apatite and HA cement |
| 82 | Verret DJ. et al. | 2005 | Retrospective study | 102 | Vault | 6 months - 6.5 years | Successful reconstruction; high osteointegration rate | 11 cases of infection or foreign body reaction requiring implant removal | HA cement |
| 83 | Iaccarino C. et al. | 2015 | Prospective study | 50 | Vault | 11 ± 7 months | Lower complication rate with alloplastic cranioplasty compared to autologous bone | 7 cases requiring reoperation (4 infections, 2 bone resorptions, 1 fracture) | HA, PMMA, PEEK |
