# Supplementary material for: Polyetheretherketone for craniomaxillofacial defects: cases report, evaluation of patients’ satisfaction and a systematic literature review
Source: Maxillofac Plast Reconstr Surg. 2025 Oct 24;47(1):36. doi: 10.1186/s40902-025-00482-9 (PMC12552202; doi:10.1186/s40902-025-00482-9)
Supplement: Supplementary file 3 — Supplementary Material 3. [file 40902_2025_482_MOESM3_ESM.docx]

Appendix C

Table B1. Statistical summary of aesthetic satisfaction by implant material

| **Material** | **Question** | **n** | **Min** | **Max** | **Mean** | **Std. Error** | **Std. deviation** |
| --- | --- | --- | --- | --- | --- | --- | --- |
| **Polyetheretherketone (PEEK)** | How would you rate your overall satisfaction with the aesthetic outcome of your surgery? | 9 | 8 | 9 | **8.67** | 0.167 | 0.500 |
|  | How satisfied are you with the symmetry of your facial features following the surgery? | 9 | 8 | 10 | **8.78** | 0.222 | 0.667 |
|  | How satisfied are you with the appearance of the surgical site (scar visibility, contour)? | 9 | 8 | 10 | **8.78** | 0.222 | 0.667 |
|  | How well do you feel the implant integrates with your natural facial structure? | 9 | 9 | 10 | **9.22** | 0.147 | 0.441 |
| **Polymethylmethacrylate (PMMA)** | How would you rate your overall satisfaction with the aesthetic outcome of your surgery? | 6 | 5 | 9 | **7.17** | 0.543 | 1.329 |
|  | How satisfied are you with the symmetry of your facial features following the surgery? | 6 | 5 | 9 | **7.67** | 0.558 | 1.366 |
|  | How satisfied are you with the appearance of the surgical site (scar visibility, contour)? | 6 | 6 | 9 | **7.83** | 0.401 | 0.983 |
|  | How well do you feel the implant integrates with your natural facial structure? | 6 | 5 | 9 | **7.67** | 0.558 | 1.366 |
| **Silicone** | How would you rate your overall satisfaction with the aesthetic outcome of your surgery? | 14 | 5 | 8 | **6.50** | 0.203 | 0.760 |
|  | How satisfied are you with the symmetry of your facial features following the surgery? | 14 | 5 | 8 | **6.57** | 0.291 | 1.089 |
|  | How satisfied are you with the appearance of the surgical site (scar visibility, contour)? | 14 | 6 | 9 | **7.50** | 0.292 | 1.092 |
|  | How well do you feel the implant integrates with your natural facial structure? | 14 | 6 | 8 | **6.71** | 0.194 | 0.726 |
| **Titanium** | How would you rate your overall satisfaction with the aesthetic outcome of your surgery? | 23 | 2 | 9 | **6.65** | 0.532 | 2.551 |
|  | How satisfied are you with the symmetry of your facial features following the surgery? | 23 | 2 | 9 | **7.09** | 0.529 | 2.539 |
|  | How satisfied are you with the appearance of the surgical site (scar visibility, contour)? | 23 | 2 | 10 | **7.43** | 0.547 | 2.626 |
|  | How well do you feel the implant integrates with your natural facial structure? | 23 | 2 | 9 | **6.96** | 0.532 | 2.549 |
